# Non-genetic stratification reveals epigenetic heterogeneity and identifies vulnerabilities of glycolysis addiction in lung adenocarcinoma subtype

**DOI:** 10.1038/s41389-022-00436-0

**Published:** 2022-10-10

**Authors:** Xuming Song, Te Zhang, Hanlin Ding, Yipeng Feng, Wenmin Yang, Xuewen Yin, Bing Chen, Yingkuan Liang, Qixing Mao, Wenjie Xia, Guiping Yu, Lin Xu, Gaochao Dong, Feng Jiang

**Affiliations:** 1 Department of Thoracic Surgery, Nanjing Medical University Affiliated Cancer Hospital & Jiangsu Cancer Hospital & Jiangsu Institute of Cancer Research, 210009 Nanjing, P. R. China; 2 Jiangsu Key Laboratory of Molecular and Translational Cancer Research, Cancer Institute of Jiangsu Province, Nanjing, P. R. China; 3 The Fourth Clinical College of Nanjing Medical University, Nanjing, P. R. China; 4 School of Basic Medicine and Clinical Pharmacy, China Pharmaceutical University, 211198 Nanjing, P. R. China; 5 Department of Cardiothoracic Surgery, The affiliated Jiangyin Hospital of Southeast University Medical College, 214400 Jiangyin, P. R. China; 6 Collaborative Innovation Center for Cancer Personalized Medicine, Nanjing Medical University, 211116 Nanjing, P. R. China

**Keywords:** Non-small-cell lung cancer, Epigenetics

## Abstract

Lung adenocarcinoma (LUAD) exhibits high heterogeneity and is well known for its high genetic variation. Recently, the understanding of non-genetic variation provides a new perspective to study the heterogeneity of LUAD. Little is known about whether super-enhancers (SEs) may be primarily responsible for the inter-tumor heterogeneity of LUAD. We used super-enhancer RNA (seRNA) levels of a large-scale clinical well-annotated LUAD cohort to stratify patients into three clusters with different prognosis and other malignant characteristics. Mechanistically, estrogen-related receptor alpha (ERRα) in cluster 3-like cell lines acts as a cofactor of BRD4 to assist SE-promoter loops to activate glycolysis-related target gene expression, thereby promoting glycolysis and malignant progression, which confers a therapeutic vulnerability to glycolytic inhibitors. Our study identified three groups of patients according to seRNA levels, among which patients in cluster 3 have the worst prognosis and vulnerability of glycolysis dependency. We also proposed a 3-TF index model to stratify patients with glycolysis-addicted tumors according to tumor SE stratification.

## Introduction

The study of tumor progression has revolutionized our understanding of tumor heterogeneity among patients [1]. The advent of genome technologies, including rapid and relatively inexpensive sequencing of cancer exomes and genomes, has enabled us to understand inter-tumoral heterogeneity, which is a mechanism of therapeutic resistance and therefore an important clinical challenge [2–4]. It has been reported that the pattern of genetic alterations in cancer driver genes in patients is highly diverse, which contributes to genetic heterogeneity [5, 6]. Dentro and colleagues determined whole-genome sequences of 2658 cancer samples across 38 cancer types, which revealed that inter-tumor heterogeneity is pervasive across cancers and each cancer displays type-specific pattern [7]. However, non-genetic changes in transcriptome, chromatin structure, and DNA accessibility of transcription factor (TF)-binding motifs are more frequent but less understood in inter-tumoral heterogeneity [8].

Non-small cell lung cancers (NSCLCs) show high heterogeneity, and have the highest mortality rate among all cancers worldwide [9]. Lung adenocarcinoma (LUAD) accounts for the majority of NSCLCs, which can be further classified into different histological subtypes depending on morphology [10, 11]. Currently, treatment decisions for individual patients with LUAD are mainly based on the characteristics of the cancer, including morphology, malignant behavior and driving molecular mutation, which contributes to tumor heterogeneity [12]. However, only a subset of LUAD cases’ evolution can be explained by genetic feature, highlighting the need of non-genetic aspects to account for heterogeneity. Emerging studies support a role for the cancer epigenome in functional tumor heterogeneity, which refers to the epigenome heterogeneity occurring by various mechanisms, such as alternative enhancer activity, different promoter hypermethylation profiles, and dynamic chromosomal accessibility [13–15].

Super-enhancers (SEs) act as cis-regulatory elements to regulate transcriptional activity in a non-genetic approach [16], but rarely explain its heterogeneity in LUAD. SEs refer to large regions of the mammalian genome with clusters of enhancers [17], which regulate abnormal global transcription activity in cancers [18, 19]. SEs have a more robust transcriptional regulatory activity than typical enhancers [20, 21]. Our group and others have found that ectopic SEs can interact with promoters of oncogenes, and thus drive abnormal gene expression, which is referred to as enhancer hijacking [22, 23]. Therefore, we hypothesize that the epigenetic heterogeneity of LUAD from the perspective of SEs may better reflect the characteristics of transcriptional regulation, which may be useful to identify its therapeutic vulnerabilities [24].

Recently, Chen et al. provided a high-resolution map for SE RNAs (seRNAs) which quantifies SE activities through the expression of conserved SEs loci [25]. However, the inter-tumor heterogeneity of SEs regulating transcription in LUAD remains uncharacterized. This study constructed a SE hetero-programming clusters (SHCs) to identify a subset of patients with glycolysis-addicted LUAD. In addition, it explained the development of glycolysis addiction in LUAD, which provides an important supplement to current therapeutic vulnerabilities of LUAD.

## Materials and methods

### Lung adenocarcinoma public data and consensus clustering

We systematically searched for publicly available LUAD seRNA expression datasets that reported full clinical annotations (especially overall survival and disease-free survival). Patients without integrated survival information were removed from further evaluation, including those in The Cancer Genome Atlas-LUAD (TCGA-LUAD) and Gene Expression Omnibus (GEO) GSE37745 datasets. The seRNA expression and SE chromatin location were downloaded from “TCGA-LUAD seRNAs in the putative SEs (n = ~200k)” [25]. The matched mRNA expression profile were downloaded using the “TCGAbiolinks” R package [26]. The pathological slides images and ATAC-seq data were downloaded from the NIH Genomic Data Commons (https://gdc.cancer.gov/). All hematoxylin and eosin (H&E) stained slides were evaluated and scored by two pathologists. Unsupervised clustering methods (K-means) for dataset analysis were used to identify enhancer activity patterns and classify patients for further analysis using the “ConsensuClusterPlus” R package [27]. TCGA-LUAD ATAC-seq bigwigs files and genomic mutation data were downloaded from Genomic Data Commons [28]. The data of H3K27ac, H3K4me1 and ERRα ChIP-seq from A549 cells (human alveolar adenocarcinoma cell line), PC-9 cells (human lung adenocarcinoma cell line) were downloaded from Encyclopedia of DNA Elements (ENCODE). These data were visualized by IGV 2.9.4 [29].

### Bioinformatics analysis

Analysis of differentially expressed genes (DEGs) was performed using the “*DESeq2*” R package [30]. The TF binding motif enrichment analysis was conducted using the “*HOMER*” software [31]. The Least Absolute Shrinkage and Selector Operation (LASSO) algorithm implemented in the “*glmnet*” R package was used to construct the “*3-TF index*” model [32]. The 22-gene “*glycolysis signature*” was used to assess glycolysis levels [33, 34].

### Clinical samples

LUAD tissue were selected randomly, from patients undergoing radical pulmonectomy and diagnosed with lung adenocarcinoma in Jiangsu Cancer Hospital affiliated to Nanjing Medical University from June 2020 to August 2020. All H&E and IHC staining slides of 12 patients selected were independently evaluated by two senior pathologists. Where the assessment was controversial, a third pathological reviewer was involved in a discussion to resolve differences. All tissues were obtained from the biobank of Jiangsu Cancer Hospital (Jiangsu Institute of Cancer Research & The Affiliated Cancer Hospital of Nanjing Medical University). All patients had signed informed consent for donating their samples. The sequences of primers for qPCR are provided in Table S1. Those primer sequences were obtained from PrimerBank(https://pga.mgh.harvard.edu/primerbank/index.html).

### Statistical analysis

The R 3.6.3 programming environment and GraphPad Prism 8.0 Software (GraphPad Software Inc., San Diego, CA, USA) were used for statistical analysis. Most graphs contain graphs for each data point and show the mean ± standard deviation. To test the significance, *t*-test, Wilcoxon rank-sum test (*n* = 3), two-sided Fishers exact test and Chi-squared test were performed, and statistically significant *p* value is indicated by an asterisk (*).

More Materials and methods are shown in Additional file 1.

## Results

### Consensus clustering for activity patterns of SEs

**Fig. 1:**
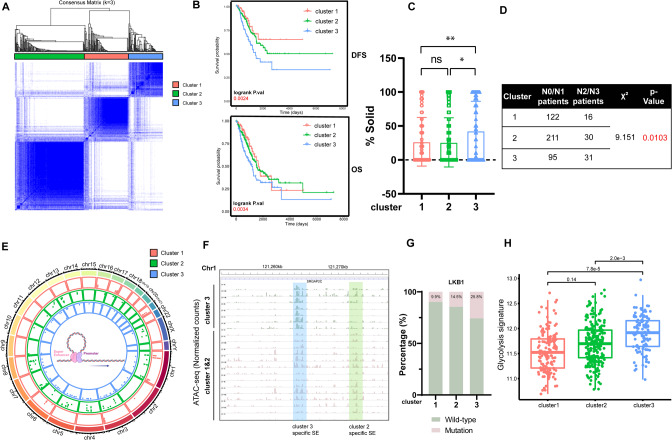
SEs hetero-programming clusters revealed various malignant characteristics. **A** Consensus clustering of seRNA expression in TCGA-LUAD datasets, proportion of samples in the three clusters. **B** Kaplan–Meier survival curves for the three clusters showing overall survival and disease-free survival. **C** Solid-subtype component percentage of whole tumor tissues for the three clusters. **D** Distant lymph node metastasis incidence in the three clusters. **E** The chromatin location of specifically activated SEs in each cluster. **F** Genome accessibility tracks for each sample of the clusters. Blue highlighting indicates cluster 3 specific SE loci. Green highlighting indicates cluster 2 specific SE loci. **G** The percentage of samples with *LKB1* mutation in each cluster. **H** The glycolysis signature assay between the three clusters. Asterisks denote statistical significance; *P < 0.0.5; **P < 0.01; ***P < 0.001.

To investigate the epigenetic heterogeneity of LUAD, tumors with qualitatively different SE activity patterns were grouped using unsupervised clustering methods [35]. To select the optimal cluster number, we assessed clustering stability using the “ConsensusClusterPlus” package (Figs. 1A, S1A, Table S2), which supported the existence of three robust SHCs in LUAD seRNA profile. Three SHCs showed significant differences in disease-free survival and overall survival (Fig. 1B). Patients in cluster 3 had worse prognosis compared with those in cluster 1 and 2. In addition, histological statistical analysis revealed that patients in cluster 1 and 2 have a greater percentage of lepidic- and acinar- subtype components, and low- or medium- risk subtypes of LUAD [36], while patients in cluster 3 have more high-risk solid- subtype components (Figs. 1C, S1B). In addition, patients in cluster 3 had a significant tendency toward advanced lymph node metastasis (N2 or above) (Fig. 1D). In the worst prognosis cluster 3 patients, these abnormally activated SE regions, partly located in 1p32.2, 17q35.3, etc., are “chromatin instability” regions which have been reported to be associated with genetic diseases and malignant tumor (Fig. 1E) [37–39]. Moreover, those regions showed increase chromatin accessibility in TCGA-LUAD patients (Figs. 1F and S1C). Overall, these results indicated that the three identified SHCs shared different clinical characteristics.

To further characterize the three identified SHCs, we analyzed their multi-omics data. We performed immune infiltration analysis according to CIBERSORTx, and found that cluster 3 showed significant aggregation in a variety of lymphocytes, including B cells, plasma cells, and macrophages (Fig. S1D). In addition, the tumor mutation burden (TMB) of cluster 3 is significantly higher (Fig. S1E). Through the analysis of the aggregation of hotspot mutations in the SHCs, we found that *EGFR*, *KRAS,* and other common driver mutations did not show significant differences between groups, apart from *LKB1* mutations, which showed significant enrichment in group 3 (Figs. 1G, S1F). It has been previously reported that tumor *LKB1* mutations are associated with cellular metabolic rearrangement, including the Warburg effect [40, 41]. We used a 22-gene glycolysis signature [34] to determine the differences in the levels of glycolysis among the three groups. The results showed that cluster 3 had a higher level of aerobic glycolysis (Fig. 1H). A hypoxia-related immunotherapeutic response score [42] showed a same tendency (Fig. S1G). Taken together, our classification based on SE activity identified three groups, and samples in cluster 3 were associated with poor prognosis and preferred glycolysis.

### Hetero-programming SE promote gene expression

**Fig. 2:**
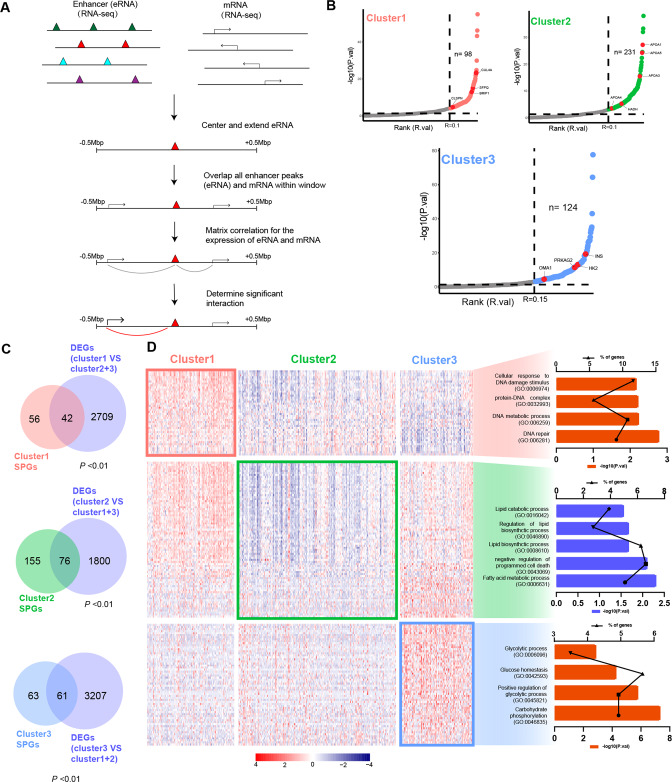
SEs hetero-programming results in enrichment of various molecular pathways. **A** Schematic of the approach used to link the activation of SEs in distal locus to mRNA through correlation of the seRNA and mRNA expression levels. **B** Two-factor plot showing mRNAs with significant correlation to nearby enhancers. **C** Venn diagrams depicting the overlap between SPGs and DEGs in each cluster. Statistical significance of the overlap between two groups of genes based on Fisher’s exact test. **D** The heatmap showing the expression of SE-regulated genes of each cluster and bar grams showing the enriched pathways by GO analysis.

SEs, as cis-acting transcriptional regulatory elements, play important roles in transcription [43]. We integrated SE regions and RNA-seq data to fully investigate the effect of specific SEs in three groups on transcriptional regulation (Fig. 2A). We used mRNAs, whose transcriptional start sites of 500 kb around SEs, and correlations between expression of seRNAs and mRNAs to define SE-regulated potential genes (SPGs) according to the reference [28]. After correlation screening, we obtained a batch of SPGs directly related to activation of SEs in the SE regulatory regions, including HK2, OMA1, PRKAG2, *etc*., which are metabolism-related genes in cluster 3 SPGs (Fig. 2B and Table S3). To investigate the transcriptional effect of hetero-programming SEs and identified hetero-hijacking mRNAs by SEs, we integrated DEGs and SEs in each cluster. Importantly, SPGs significantly overlapped with DEGs (activated in clusters 1 &3, repressed in cluster 2), referred to as SE-regulated genes (Fig. 2C and Table S4–5). Gene ontology (GO) analysis of the SE-regulated genes in each SHC showed that DNA-repair associated pathways were activated in cluster 1, lipid metabolism associated pathways were inhibited in cluster 2, and glycolysis associated pathways were activated in cluster 3, which is consistent with the aforementioned. These results indicated that abnormally activated SE in cluster 3 promoted transcriptional regulation-dependent malignant progression.

### Differential TF-SE interactions correspond with glycolysis reprogramming

**Fig. 3:**
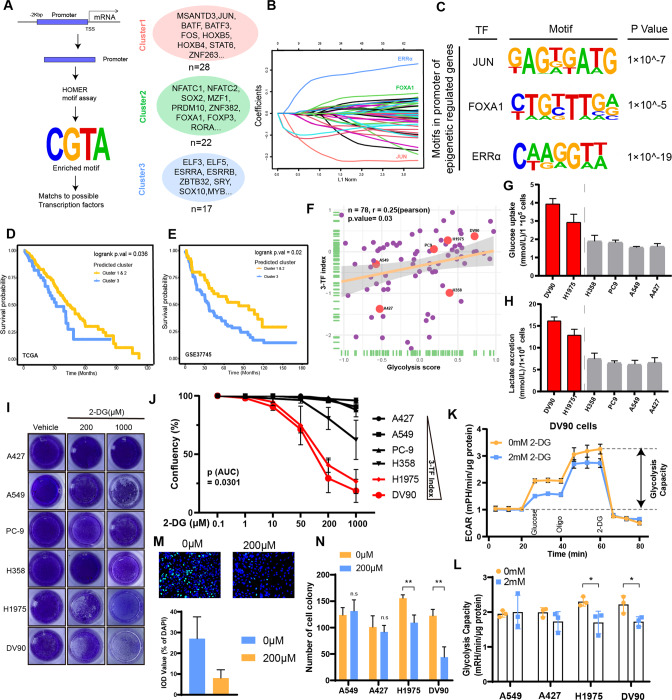
Differential TF-SE interactions correspond with glycolysis reprogramming. **A** Schematic of the approach used to identify TFs with potential molecular function. **B** The LASSO coefficient profiles for the prediction of SHCs. **C** The motifs in the promoters of SE-regulated genes. **D**, **E** Kaplan–Meier survival curves for predicted clusters (according to the 3-TF index model) displaying overall survival in TCGA cohort and GSE37745. **F** Correlation plots showing the correlation between glycolysis score and 3-TF index value in 78 LUAD cell lines from the CCLE database. **G**, **H** There were tendencies of high glucose uptake and lactate excretion in cell lines with high 3-TF index than in cell lines with low 3-TF index. **I**, **J** Effect of 2-DG on the confluence of LUAD cell lines with high 3-TF index and low 3-TF index. Extracellular flux assays using Seahorse (**K**, **L**), EdU assay (**M**) and clone formation assay (**N**) showing that cell lines with high 3-TF index (DV90, H1975) have higher 2-DG sensitivity than cell lines with low 3-TF index (A549, A427). Asterisks indicate statistical significance; *P < 0.0.5; **P < 0.01; ***P < 0.001.

Cooperative TFs are required for SE activity and transcriptional regulation [43, 44]. To investigate the molecular mechanism underlying SE hetero-programming in each SHC, we first performed *de novo* motif searches for promoters of SE-regulated genes, and identified 29, 22, and 17 TFs footprints in clusters 1, 2 and 3, respectively (Fig. 3A and Table S6). Then, to obtain core TFs for the malignant phenotype, we used a Least Absolute Shrinkage and Selector Operator (LASSO) model [45], which identified *JUN* (cluster 1), *FOXA1* (cluster 2) and *ERRα* (cluster 3) as core TFs in each cluster (Fig. 3B, C and Table S6). A 3-TF index model was constructed according to the expression and weights of the core TFs (Fig. S2A), which showed considerable effective survival prediction and cluster prediction capability in TCGA-LUAD (Fig. 3D and Fig. S2B). In addition, survival predictions of the 3-TF index model were validated in GSE37745 dataset (Fig. 3E), which suggested that the 3-TF index model has robust stratification concordance with SHCs. Overall, we constructed the 3-TF index model as a general method to predict the classification of patients with different outcomes.

Next, to confirm our findings in LUAD immortalized cells, we used the 3-TF index model to evaluate LUAD cell lines from the Cancer Cell Line Encyclopedia (CCLE). Remarkably, we found that the 3-TF index values of the cell lines were consistent with the 22-gene glycolysis signature (Fig. 3F and Table S7). To further confirm the correlation of core transcription factor activity with the level of aerobic glycolysis in candidate LUAD cell lines. We designed a transcriptional factor *ERRα* ability luciferase reporter plasmid (Fig. S2C upper). The transcriptional factor ability of *ERRα* were tested in 6 LUAD cell lines, respectively. The results showed that cell lines with high Glycolysis score (including DV90 and H1975) have higher transcriptional factor ability of *ERRα* than cell lines with low Glycolysis score (including A549, PC9, H358 and A427) (Fig. S2C bottom). These above results verify the ability of core transcription factors to regulate the glycolytic activity of tumor cells. Assay of glucose uptake and lactate excretion revealed the cell lines with high 3-TF index (including DV90 and H1975) had a higher level of aerobic glycolysis than cell lines with low 3-TF index (cluster 1&2, including A549, PC-9, A427 and H358) (Fig. 3G, H). We used 2-DG, a hexokinase inhibitor and glucose analog, to test the sensitivity of different cell lines to glycolytic pathways. It inhibited the confluence (Fig. 3I, J), glycolysis capacity (Fig. 3K, L), and malignant progression (Figs. 3M, N and S2D-E) of cell lines with high 3-TF index to a greater extent than those of the cell lines with low 3-TF index. These findings suggested that increased glycolysis in cluster 3-like samples, which may confer a therapeutic vulnerability to glycolytic inhibitors.

### ERRα regulates aerobic glycolysis and malignant progression

As ERRα was found to play a core role in SE reprogramming in cluster 3, which has also been reported as a risk factor in multiple tumors [46–49], we asked whether ERRα contributed to glycolysis and malignant progression in cluster 3 cell lines. Both siRNA and overexpression plasmids, which we used in subsequent studies, can significantly regulate the expression of ERRα (Fig. S2F–G). The assay of glucose uptake and lactate excretion revealed that glycolysis mediated by ERRα in cluster3-like cell lines (DV90 and H1975), while in the non-cluster3-like cell line (A549), those above changes were exhibited (Fig. S3A). In addition, the extracellular flux assays revealed that the glycolysis capacity of the cells was significantly inhibited after knocking down of ERRα, in DV90 (Fig. S3B). In addition, the malignant progression indicators, including proliferation rate (Fig. S3C–E), invasion and migration abilities (Fig. S3F), were found to be positively mediated by ERRα in cluster3-like cell lines. While, the non-cluster3-like cell line did not show above changes significantly. These findings indicate that the aerobic glycolysis and malignant progression of the cluster 3 cell lines are mediated by ERRα, but had no significant effect on non-cluster3-like cell lines. TFs may play a role of cofactor for SE hijacking oncogenes’ promoter and promote SE remodeling, which has been reported in previous studies [50]. Thus, we propose an axis in which the hetero-programming SE in cluster 3 hijacks the oncogenes’ promoters through the assistance of ERRα, which ultimately promotes the aerobic glycolysis and malignant progression of the tumor.

### SE hijacks the HK2 promoter and regulates its transcriptional activity assisted by ERRα

**Fig. 4:**
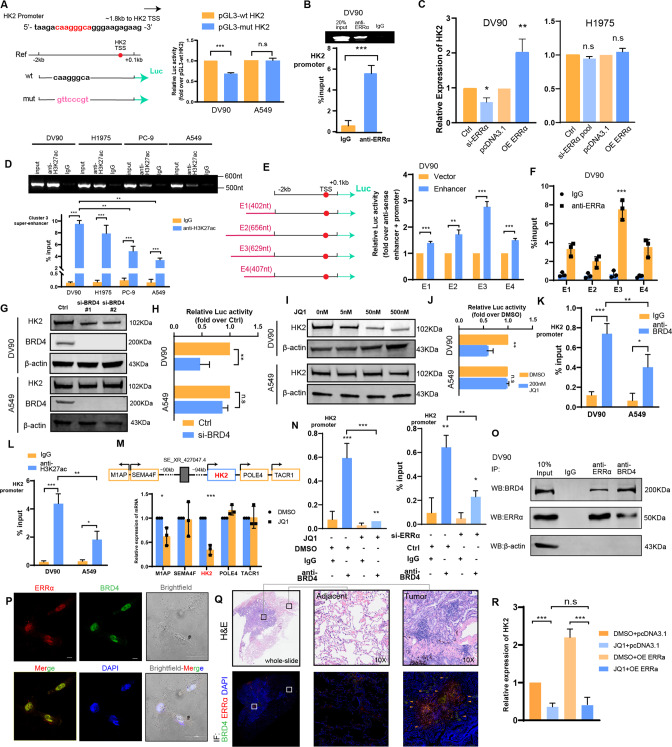
SE hijacks the HK2 promoter and regulates its transcriptional activity assisted by ERRα. **A** The DV90 and A549 cell lines were transfected with the indicated plasmids for 48 h, respectively. The levels of luciferase activity were normalized to the pRL-TK luciferase activity. **B** DV90 cells were subjected to ChIP analysis using an anti-ERRα antibody and quantified by qPCR analysis of the HK2 promoter region. **C** The mRNA expression level of HK2 regulated by ERRα in DV90 and A549 cell lines. **D** Four cell lines with different 3-TF index values were subjected to ChIP analysis using an anti-H3K27ac antibody and quantified by qPCR analysis of the SE_XR_427047.4 locus. **E** The luciferase activity of four enhancer elements was measured by a dual-luciferase reporter assay in DV90 cells. **F** DV90 cells were subjected to ChIP analysis an anti-ERRα antibody and quantified by qPCR analysis of four enhancer elements. The protein expression (**G**) and transcription activation (**H**) of HK2 regulated by interfering with BRD4 in DV90 and A549 cell lines. JQ1 disrupted the protein expression (**I**) and transcription activation (**J**) of HK2 in DV90 and A549 cell lines. Cluster 3-like (DV90) and non-cluster 3-like (A549) cells were subjected to ChIP analysis using an anti-BRD4 antibody (**K**) and an anti-H3K27ac antibody (**L**). The association with the promoter region of HK2 was quantified by qPCR analysis. **M** The expression of mRNAs nearby the SE_XR_427047.4 locus after treated with or without 200 nM JQ1 for 24 h. **N** DV90 cells were treated with or without 200 nM JQ1 for 24 h. The cells were subjected to ChIP analysis using an anti-BRD4 antibody and an anti-H3K27ac antibody. The association with the promoter region of HK2 was quantified by qPCR analysis. **O** Immunoprecipitation with antibodies against ERRα, BRD4, or IgG followed by Western blot analysis was performed for the indicated proteins. The Immunofluorescence staining for ERRα and BRD4 performed on DV90 cells (**P**) and LUAD tissue slide, three independent experiments were performed on three slides from different LUAD patients (**Q**). **R** qRT-PCR analysis revealing that the ERRα-regulated expression of HK2 partly depends on SEs. Asterisks indicate statistical significance; *P < 0.0.5; **P < 0.01; ***P < 0.001.

HK2 is necessary for accelerating glucose flow, tumor initiation, and maintenance [51]. Due to the high glycolysis level in cluster 3 patients, we selected HK2 from the cluster 3 SE-regulated genes as an example. The mRNA and protein levels were increased both in in vitro experiment and CCLE transcription data (Fig. S4A and Table S7). Moreover, 91 lung NSCLC cancer cell lines interrogated by genome-wide loss-of-function CRISPR screening [52] revealed that HK2 dependency is greater in tumors with high ERRα dependency than in those with low ERRα dependency (Fig. S4B). We found an ERRα binding motif-like sequence located ~1.8 kb upstream of the HK2 transcription start sequence (TSS). Based on the wild-type sequence including conserved ERRα-binding motif(pGL3-wt-HK2), we generated a mutant luciferase reporter with the anti-sense sequence of the ERRα-binding motif (pGL3-mut-HK2), and used those to perform dual-luciferase assays (Fig. 4A, left). The results showed that the pGL3-mut-HK2 clearly decreased the induction of luciferase activity in DV90 and H1975 cells, while no significant changes in A549 (Fig. 4A, right and Fig. S4C). In addition, the ChIP-PCR assay revealed that ERRα occupied the promoter of the HK2 gene (Figs. 4B and S4D). Also, the expression of HK2 was regulated by ERRα both at the mRNA and protein levels in DV90 and H1975 (Figs. 4C and S4E–F). These results suggested that ERRα promoted the transcriptional activity of HK2 as a TF in cluster3-like cell lines, rather than non-cluster3-like cell lines.

We further explored whether ERRα plays a transcriptional regulatory role by assisting a nearby cluster 3 specific activated SE (SE_XR_427047.4). We analyzed the H3K27ac level and chromatin accessibility of the SE_XR_427047.4 locus. The DV90 and H1975 cells (high 3-TF index value, cluster 3-like) showed higher H3K27ac level and chromatin accessibility than the PC-9 and A549 cells (low 3-TF index value, non-cluster 3-like) (Figs. 4D and S4G). The public anti-H3K27ac and anti-H3K4me1 ChIP-seq data indicated that although the SE_XR_427047.4 activity was sharply increased in cluster 3 samples, they were all more active in tumors (Fig. S4H). We also divided the SE_XR_427047.4 into 4 components (Fig. 4E, left and Fig. S4H), and constructed plasmids with dual-luciferase reporter genes, containing E1-E4 and the promoter of HK2. Strong transcription-enhancing activity was observed in cells transfected with enhancer plasmids compared to control plasmids, especially those transfected with the E3 plasmid (Fig. 4E, right). Furthermore, ChIP-qPCR assay with anti-ERRα revealed a significantly higher enrichment in E3 region (Fig. 4F). These results suggested that hetero-programming SE_XR_427047.4 in cluster 3 leads to the hijacking of the promoter of HK2 and regulates its transcriptional activity.

**Fig. 5:**
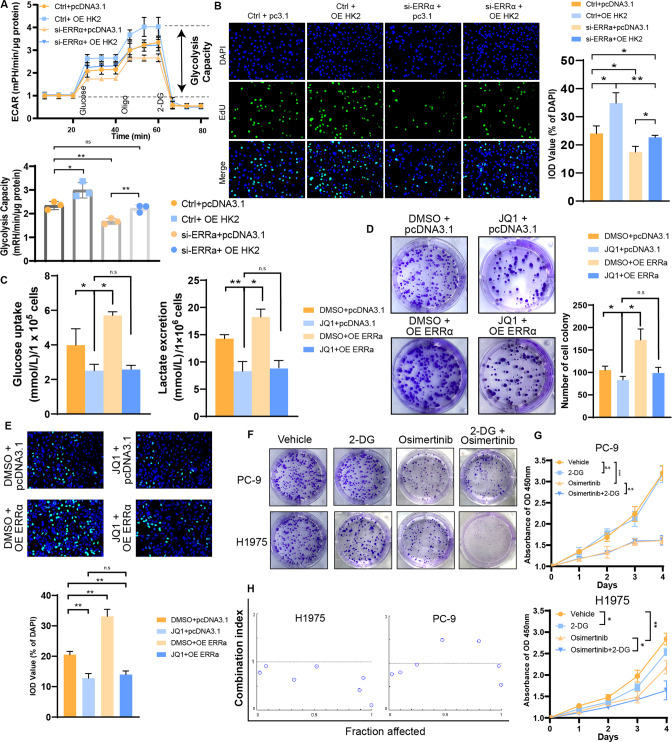
ERRα assists SE to regulate aerobic glycolysis and malignant progression. **A** Extracellular flux assays showing that ERRα mediated aerobic glycolysis partly depends on regulating HK2 in DV90 cell line. **B** EdU assays showing that ERRα mediated malignant progression partly depends on regulating HK2 in DV90 cell line. Glucose uptake, lactate excretion (**C**), clone formation assay (**D**) and EdU assay (**E**) showing that ERRα mediated malignant progression partly depends on regulating SEs in DV90 cell line. Clone formation assay (**F**) and CCK-8 assay (**G**) revealing the inhibitory efficacy of 2-DG, osimertinib and their combination in the EGFR^mut^ cluster 3-like cell line (H1975) and EGFR^mut^ non-cluster 3-like cell line (PC-9). **H** Combination index of 2-DG and osimertinib in the indicated cell lines. Combination index > 1 indicated antagonism, combination index < 1 indicated synergy.

Previous studies identified BRD4 as a bromodomain and extra-terminal domain (BET) protein family member, which binds acetylated H3K27 at promoters as well as SEs, conjugates them together and mediates transcriptional co-activation and elongation [53, 54]. JQ1, a small-molecule inhibitor preferentially blocks the binding between BRD4 and SEs, which diminishes the expression of SE target genes [55]. Accordingly, we further explored whether HK2 expression was regulated by BRD4. The result showed that HK2 expression was suppressed when BRD4 is repressed (Figs. 4G and S5A), as well as HK2 transcriptional activation in DV90 (Fig. 4H). However, no significant changes were obtained in A549. These results were confirmed in an experiment showing the effect of JQ1 in a dose-dependent manner (Figs. 5I, J and S5B). The ChIP-qPCR assay with anti-BRD4 in DV90 cells showed significant enrichment located in the HK2 promoter, which was co-occupied by the acetylated H3K27 signal (Figs. 4K–L and S5C). However, those enrichment were greater in cluster 3-like cells than other cells (Figs. 4K, L and S5D). In addition, after treatment with JQ1, the expression of HK2 decreased significantly instead of genes nearby (Fig. 4M), which indicated that the transcriptional activation of HK2 regulated by SE_XR-427047.4 is specific.

Moreover, the BRD4 binding described above was reversed after treated with JQ1, a similar trend was also observed after ERRα knockdown (Fig. 4N). Given the binding of ERRα to chromatin within the E3 region (Fig. 4F), we infer that ERRα assisted SE in hijacking the HK2 promoter by cooperating with BRD4 to promote HK2 transcriptional regulation. The co-immunoprecipitation assay revealed the interaction between BRD4 and ERRα (Fig. 4O), which was also confirmed by demonstrating colocation by an immunofluorescence assay in DV90 (Fig. 4P). Moreover, immunofluorescence of ERRα and BRD4 in tissue sections of 3 patients with lung adenocarcinoma also showed that they were widely co-localized in tumor tissues, but isolated from each other in adjacent tissues (Figs. 4Q and S5E). In addition, after suspended SE-promoter loop using JQ1, the ERRα -regulated HK2 expression was reversed (Figs. 4R and S5F). In short, ERRα assists SE in hijacking the promoter of HK2 and regulates its transcriptional activity by cooperating with BRD4.

### ERRα assists SE in mediating aerobic glycolysis and malignant progression

We further explored that glycolysis and malignant progression are driven by ERRα and HK2. Using DV90 cell line as the in vitro model for cluster3-like sample, extracellular flux assays revealed that the glycolysis capacity was significantly decreased by ERRα knockdown, which was reversed by HK2 overexpression (Fig. 5A). Expectedly, receding of malignant proliferation by knockdown ERRα was reserved by overexpression HK2 (Fig. 5B). We also investigated whether ERRα-driven glycolysis and malignant progression were partly dependent on SE hijacking. Global SE-promoter loop inhibition significantly decreased the glycolysis capacity (Fig. S6A–C). In addition, the regulation of cancer cells glycolysis capacity and malignant proliferation by ERRα partly depends on the SE-promoter loops (Fig. 5C–E), which indicated that ERRα acted as a cofactor for SE hijacking.

Previous studies showed that a clinically significant proportion of patients with LUAD had epidermal growth factor receptor (EGFR) mutations [56]. EGFR activating mutations predict sensitivity to EGFR tyrosine kinase inhibitors (TKIs), especially in third-generation EGFR-TKIs, such as Osimertinib [57]. In the clinical treatment of advanced LUAD, Osimertinib is often used in combination with other drugs to inhibit the progression of malignant tumors [58]. Previous studies have shown that enhanced glycolysis is the key to maintaining the stability of EGFR [59], which suggests that the combined inhibition of the glycolysis pathway and EGFR pathway may produce better therapeutic effects. We further investigated whether combining EGFR-TKIs and glycolytic inhibitors could inhibit EGFR^mut^ in cluster 3-like LUAD cell lines more effectively than monotherapy. We selected the PC-9 cell line as the EGFR^mut^ non-cluster 3 cell line and the H1975 cell line as the EGFR^mut^ cluster 3 cell line. In vitro experiments revealed that, although Osimertinib significantly suppresses proliferation of malignant cells, the potency of 2-DG or combination was quite slight in PC-9 cells. However, the Osimertinib plus 2-DG combination showed a stronger potency than any of the monotherapies in H1975 (Figs. 5F, G and S6D). The Chou-Talalay combination index model was used to determine the synergistic anti-tumor effect of 2-DG with Osimertinib in PC-9 and H1975 cells. We found that the combination index values were <1 in H1975 cells but not in PC-9, suggesting a synergistic effect between the glycolysis inhibitor and Osimertinib in cluster 3 LUAD (Fig. 5H). These results indicated that glycolysis confers a therapeutic vulnerability to patients with EGFR^mut^ in cluster 3, that may benefit from the combination of glycolysis inhibitors and EGFR-TKIs.

### Core TFs expression indicated the glycolysis capacity in LUAD patients

**Fig. 6:**
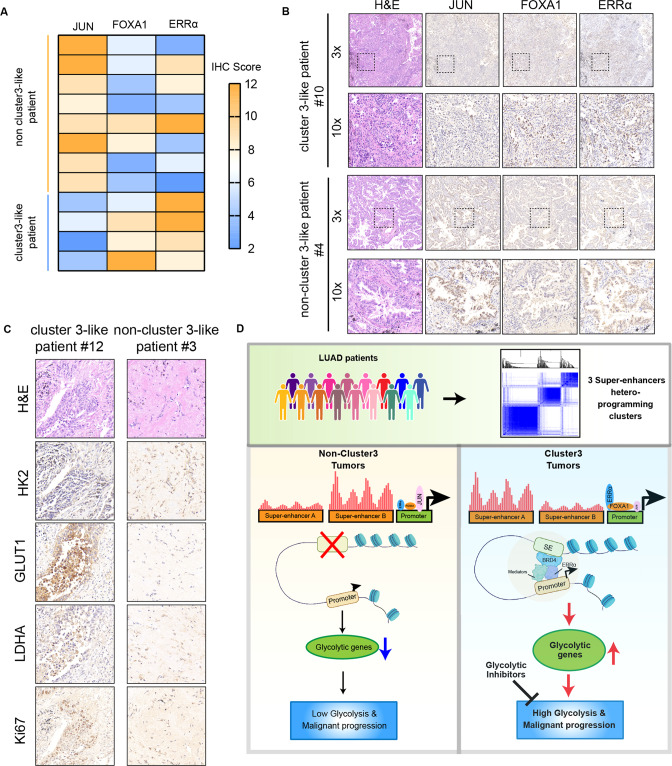
Core TFs expression indicated the glycolysis capacity in LUAD patients. **A** Heatmap displaying the IHC scores of 3 core TFs expression in LUAD samples from the Jiangsu Cancer Hospital, with a representative image shown in (**B**). **C** Representative IHC staining image of HK2, GLUT1, LDHA and Ki67 in LUAD samples from the Jiangsu Cancer Hospital. **D** We used a large-scale clinical seRNA expression profile cohort and unsupervised clustering to obtain the three clusters. Through the enrichment of TF motifs, we identified the core TFs that regulate the transcription of each cluster, namely ERRα, FOXA1 and JUN. We confirmed that ERRα in cluster 3 can act as a cofactor of BRD4 to assist SE-promoter loops in SE hijacking, activating glycolysis-related target gene expression, and promoting glycolysis and malignant progression of tumor cells.

We measured the mRNA and protein expression levels of the three core TFs in 12 LUAD patients, and found that, as expected, their expression levels were positively correlated (Table S8 and Fig. S6E). We divided those patients based on the expression levels of the core TFs in those 12 LUAD tissues (Figs. 6A, B and S6F). Consistent with the previous results, cluster 3-like tumors showed increased expression of several glycolysis markers, including *HK2*, *GLUT1,* and *LDHA*, as well as proliferation marker, Ki67 (Figs. 6C and S6G). These findings confirms that cluster 3 LUADs with ERRα^+^ FOXA1^-/+^ JUN^-^ have great glycolysis and proliferation capacity, as the potential target of of glycolysis inhibitors.

## Discussion

This study used a large-scale clinical seRNA expression profile cohort and unsupervised clustering to identify three clusters with different prognosis, as well as the tumor microenvironment composition, and transcriptome characteristics in LUAD. Integrative analysis of SE region and RNA-seq data, we found that cluster 3 samples have more glycolytic characteristics. Through the enrichment of TF motifs, we identified the core TFs that regulate the transcription of each cluster, namely ERRα, FOXA1 and JUN. We confirmed that ERRα in cluster 3 can act as a cofactor of BRD4 to assist SE-promoter loops in SE hijacking, activating glycolysis-related target gene expression, and promoting glycolysis and progression of malignant tumor cells (Fig. 6D), which confers a therapeutic vulnerability to patients in cluster 3.

Recently, studies have focused on epigenetic alterations in LUAD progression to investigate epigenomic heterogeneity. Yan et al. investigated the H3K27ac histone modification profiles of tumors and adjacent normal lung tissues, and defined two LUAD subgroups with significantly different prognosis, according to the intertumoral variability of H3K27ac levels at gene promoters and distal enhancers [15]. It has also been reported that epigenetic and transcriptional reprogramming reshape histological features of LUAD from indolent to aggressive patterns, thus contributing to morphological intratumor heterogeneity, which is not driven by genetic alterations [60]. In this study, we used seRNA levels reflecting the activity of SEs to classify three clusters of LUAD patients, cluster 1, cluster 2, and cluster 3, and we also found that patients in cluster 3 have significantly worse prognosis than those in cluster 1 and 2. We also found that cluster 3 patients tend to have more high-risk solid-subtype components and advanced lymph node metastases, which are associated with poor outcomes [61, 62]. Notably, we found no significant associations between the three clusters and genetic features, except for *LKB1* mutations, which was enriched in cluster 3. In recent study, *LKB1* has been identified as a master regulator of chromatin accessibility, which leads to differential epigenetic reprogramming [8]. However, very little is known about whether *LKB1* mutation directly results in epigenetic heterogeneity, which should be further explored in the future.

Aerobic glycolysis is widely known as a hallmark of malignant tumors [63]. Lung-specific loss of histone methyltransferase KMT2D widely impairs epigenomic signals for SEs/enhancers to promote glycolysis, ultimately resulting in lung tumorigenesis [64]. It has also been reported that cancer cells addicted to tyrosine kinase inhibitors (TKI) displayed a metabolic shift toward increased glycolysis and lactate production, which induced cancer-associated fibroblasts to produce hepatocyte growth factor (HGF) to activate MET-dependent signaling in cancer cells, and ultimately sustained resistance to TKIs [65]. Besides cancer cells dependent on glycolysis, macrophages in the pre-metastatic niche phagocytose tumor-derived exosomes to become polarized towards an immunosuppressive phenotype through NF-kB-dependent, glycolytic-dominant metabolic reprogramming [66]. In this study, we focused on patients in cluster 3 with the worst prognosis, and found that these patients are in a glycolysis-dependent state. The DV90 and H1975 cell lines with high glycolysis score are more sensitive to 2-DG, a hexokinase inhibitor and glucose analog, than the cell lines with low glycolysis score. Notably, we found that tumors of cluster 3 patients are infiltrated with more macrophages than those of cluster 1 or cluster 2 patients, which suggests that whether macrophages promote glycolysis of cancer cells should be explored. We also found that treatment with a combination of Osimertinib plus 2-DG combination had a strong effect on EGFR mutant cluster 3 like cell line H1975, with a combination index value of <1. Further studies should be performed to explore potential molecular mechanism of this synergistic effect and validated in large cohorts.

In this study, we have determined that ERRα can regulate the transcription of glycolysis-related genes, including HK2, by assisting SE, thereby inducing glycolysis and malignant phenotypes. In addition, LUAD patients can be classified into cluster 3-like and non-cluster 3-like patients by IHC analysis of ERRα, FOXA1 and JUN. Orphan receptors, such as ERRα, have been established as major receptors of energy metabolism, are ubiquitous and enriched in metabolically active tissues [67], including malignant tumors. It has been previously reported that ERRα induces GCK transcription to promote glucose phosphorylation and stimulate glycolysis in liver tissues [68]. Moreover, ERRα has also been reported to be involved in HIF-induced glycolysis gene expression under hypoxic conditions [69]. To the best of our knowledge, this is the first time that ERRα has been shown to be partly dependent on the regulation of SEs for glycolysis and malignant progression of LUAD. Remarkably, ERRα was also found to increase A20 expression by binding its promoter and inhibiting M1 macrophages [70]. These studies suggest a possibility for ERRα to promote M2 macrophages polarization in cluster 3 LUAD patients, which should be explored in the future.

This study describes a classification according to LUAD SEs hetero-programming to predict the prognosis of patients. We established that SEs hijacking events are assisted by core TFs to regulate cluster-specific genes expression. Our data further identified ERRα-assisted cluster 3 LUADs as important markers of vulnerability to glycolysis inhibitors.

## Supplementary information

Additional file 1

Additional file 2

Additional file 3

## Data Availability

The seRNA profile were downloaded from The Cancer eRNA Atlas (https://bioinformatics.mdanderson.org/public-software/tcea/). The source data of other figures are provided as a Source Data file. All other data are available from the authors upon reasonable requests.
