# Supplementary material for: Non-genetic stratification reveals epigenetic heterogeneity and identifies vulnerabilities of glycolysis addiction in lung adenocarcinoma subtype
Source: Oncogenesis. 2022 Oct 10;11(1):61. doi: 10.1038/s41389-022-00436-0 (PMC9550819; doi:10.1038/s41389-022-00436-0)
Supplement: Supplementary file 1 — Additional file 1 [file 41389_2022_436_MOESM1_ESM.docx]

**Title page**

**Non-genetic stratification reveals epigenetic heterogeneity and identifies vulnerabilities of glycolysis addiction in lung adenocarcinoma subtype**

**Running title:** **Epigenetic stratification shows glycolysis addiction**

**Additional file 1 Extended Methods**

**Extended Methods**

1. Public cell line data analysis, cell culture and treatment

The cancer cell line annotations and mRNA expression level data were downloaded from the Cancer Cell Line Encyclopedia (CCLE). All cell lines used in this study (*DV90, NCIH1975, NCIH358, A549, PC-9, A427*) were purchased from Shanghai Institute of Cell Biology, Chinese Academy of Sciences (Shanghai, China), and identified by short tandem repeat sequence analysis. All cells were cultured in a humidified environment at 37℃ with 5% CO_2_.

2. Glucose uptake and lactate excretion assay

Cells were counted on a *Countess 3 FL Automated Cell Counter* (Thermo Fisher Scientific Inc., Waltham, MA, USA) before seeding in a 96-well plate. Glucose uptake and lactate excretion assays were conducted using the Glucose Uptake Assay Kit (#ab136955; Abcam, Cambridge, UK) and Lactic Acid LD Assay Kit (#KGT023; KeyGEN BioTECH, Nanjing, China). Results represent the mean ± standard deviation (SD) of three experiments.

3. Inhibitor Experiments

Cells were seeded at a density of 1× 10^4^ cells per well in 96-well plates. Cells were cultured in medium containing 2-deoxy-D-glucose (2-DG; #S4701; 0 to 1000 μM; Selleck Chemicals, Houston, TX, USA) for 7 days. Then, the cells were stained with crystal violet and quantified using the ImageJ software(14). The BET bromodomain inhibitor JQ1 (#S7110; Selleck Chemicals), dissolved in DMSO, was mixed with the medium (final concentration 200 nM) and used to treat cells for 24 h(35).

4. 5-Ethynyl-2'-deoxyuridine (EdU) assay

Cells were seeded in 96-well plates and subjected to corresponding treatment. Subsequently, cells were incubated with 50 mM EdU (#KGA331; KeyGEN BioTECH) for 2 hours, fixed with 4% paraformaldehyde, and incubated with Apollo Dye Solution to label proliferating cells. Cell nuclei were then counterstained with 4′,6-diamidino-2-phenylindole (DAPI). Proliferating cells with green fluorescent signal were visualized by fluorescence microscopy on a *Leica DM4000 B LED fluorescence microscope* (Leica Microsystems GmbH, Wetzlar, Germany). Results represent the mean ± standard deviation (SD) of three experiments.

5.Clone formation assay

400 cells per well were seed on 6-well plates and 200 cells per well for 12-well plates. Clones are harvested in 10 days. The clones were stained with 1% crystal violet.

6. Cell proliferation analysis with the counting kit-8 (CCK8) assay

The cell proliferation analyses were performed using a CCK-8 cell proliferation assay Kit (#KGA317; KeyGEN BioTECH,) following the manufacturer’s instructions. All assays were performed in triplicate. Results represent the mean ± standard deviation (SD) of three experiments.

7. Extracellular flux assays

The extracellular acidification rate (ECAR) in cell lines was measured using the Seahorse XF96e Extracellular Flux Analyzer (Agilent Technologies Inc., Santa Clara, CA, USA). The ECAR, used to assess key parameters of glycolytic flux (*e.g.*, basal glycolysis and glycolytic capacity), was measured by the sequential additions of glucose, oligomycin, and 2-DG in the Seahorse Analyzer using the Glycolysis Stress Test Kit (#103020-100; Agilent Technologies Inc.) according to the manufacturer’s protocol. All results were normalized to the protein concentrations using a bicinchoninic acid (BCA) Protein Assay Kit (#P0012; Beyotime, Shanghai, China). Results represent the mean ± standard deviation (SD) of three experiments.

8. Cell transfection

The small interfering RNAs (siRNAs) targeting ERRα and BRD4 were purchased from BIOGOT (Nanjing, China). The ERRα -overexpressing plasmid and HK2-overexpressing plasmid, which were synthesized by BIOGOT, were constructed by sequencing synthesis and subcloned into pcDNA3.1(+) (Public Protein/Plasmid Library, Nanjing, China). Plasmid transfection was performed using Lipofectamine 3000 Transfection Reagent (#L3000075; Thermo Fisher Scientific Inc.). Transient transfection of siRNA was performed using Lipofectamine RNAiMAX (#LMRNA015; Thermo Fisher Scientific Inc

9. Dual-Luciferase reporter assay

The HK2 promoter ERRα binding motif wild-type plasmid, mutated plasmid, enhancer (E1-E4) plus HK2 promoter plasmid and antisense enhancer (E1-E4) plus HK2 promoter plasmid were inserted into the pGL3 basic vector (Promega Corporation, Madison, WI, USA). All were co-transfected with a pRL-TK plasmid into cells by using Lipofectamine 3000 (Thermo, #L3000075) in triplicate. Luciferase activity was measured using the Dual-Glo Luciferase Assay System (Vazyme, #DD1205-01) according to the manufacturer’s guidelines. Results represent the mean ± standard deviation (SD) of three experiments.

10. RNA extraction and quantitative real-time polymerase chain reaction qPCR analysis

The q-PCR analysis was performed using the PrimeScript™ RT Master Mix (#RR036A; Takara Bio Inc., Shiga, Japan) and [PowerUp™ SYBR™ Green](https://www.thermofisher.cn/order/catalog/product/cn/zh/A25741) Mix (#A25742; Applied Biosystems Corporation, Foster City, CA, USA) on the Applied Biosystems 7500 Real-Time PCR system (Applied Biosystems Corporation) to quantitatively measure the expression of RNA. All reactions were performed in triplicate. The ACTB mRNA was used as internal control of mRNA. Results represent the mean ± standard deviation (SD) of three experiments. The primer sequences are provided in Table [S1](https://www.ncbi.nlm.nih.gov/pmc/articles/PMC8743672/#jcmm17097-sup-0006). Those primer sequences were obtained from PrimerBank(https://pga.mgh.harvard.edu/primerbank/index.html).

11. Western blot analysis and [immunofluorescence](javascript:;) analysis

Western blot analysis and [immunofluorescence](javascript:;) analysis were performed according to standard protocols. Anti‐β-actin(#3700), anti-ERRα(#13826), anti-HK2(#2867), anti-BRD4(#13440) were purchased from Cell Signaling technology Inc. (CST Inc., Danvers, MA, USA). The immunofluorescence images were acquired by confocal laser scanning microscopy using a Nikon A1R SI Confocal Laser Point Scanning System (Nikon Instruments Inc., Tokyo, Japan).

12. Chromatin immunoprecipitation-quantitative real-time PCR (ChIP-qPCR)

ChIP was performed according to the manufacturer’s instructions using the Magna ChIP A kit (#17-610; MilliporeSigma, Burlington, MA, USA,). Anti-BRD4, anti-ERRα, anti-H3K27ac (#4353) were purchased from CST Inc. The qPCR analysis was performed as described above. Results represent the mean ± standard deviation (SD) of three experiments.

13. Immunohistochemistry (IHC) analysis

LUAD tissues were fixed with 10% formalin and embedded in paraffin. Subsequently, the Tissue samples were cut into 5-μm-thick sections and then individually incubated overnight with primary antibodies against ERRα(#13826), Ki-67(#9449S), HK2(#2867), GLUT1(#73015S), LDHA(#3582S) (CST Inc.), FOXA1 (#20411-1-AP, Proteintech), JUN (#66313-1-Ig, Proteintech). The sections were subsequently incubated with anti-horseradish peroxidase (HRP)-polymer-conjugated secondary antibody (CST Inc.) at 37 °C for 1 h and stained with a 3,3-diaminobenzidine (DAB) solution.
