# Supplementary material for: Non-genetic stratification reveals epigenetic heterogeneity and identifies vulnerabilities of glycolysis addiction in lung adenocarcinoma subtype
Source: Oncogenesis. 2022 Oct 10;11(1):61. doi: 10.1038/s41389-022-00436-0 (PMC9550819; doi:10.1038/s41389-022-00436-0)
Supplement: Supplementary file 2 — Additional file 2 [file 41389_2022_436_MOESM2_ESM.docx]

**Title page**

**Non-genetic stratification reveals epigenetic heterogeneity and identifies vulnerabilities of glycolysis addiction in lung adenocarcinoma subtype**

**Running title:** **Epigenetic stratification shows glycolysis addiction**

**Additional file 2 Figure S1-S6**


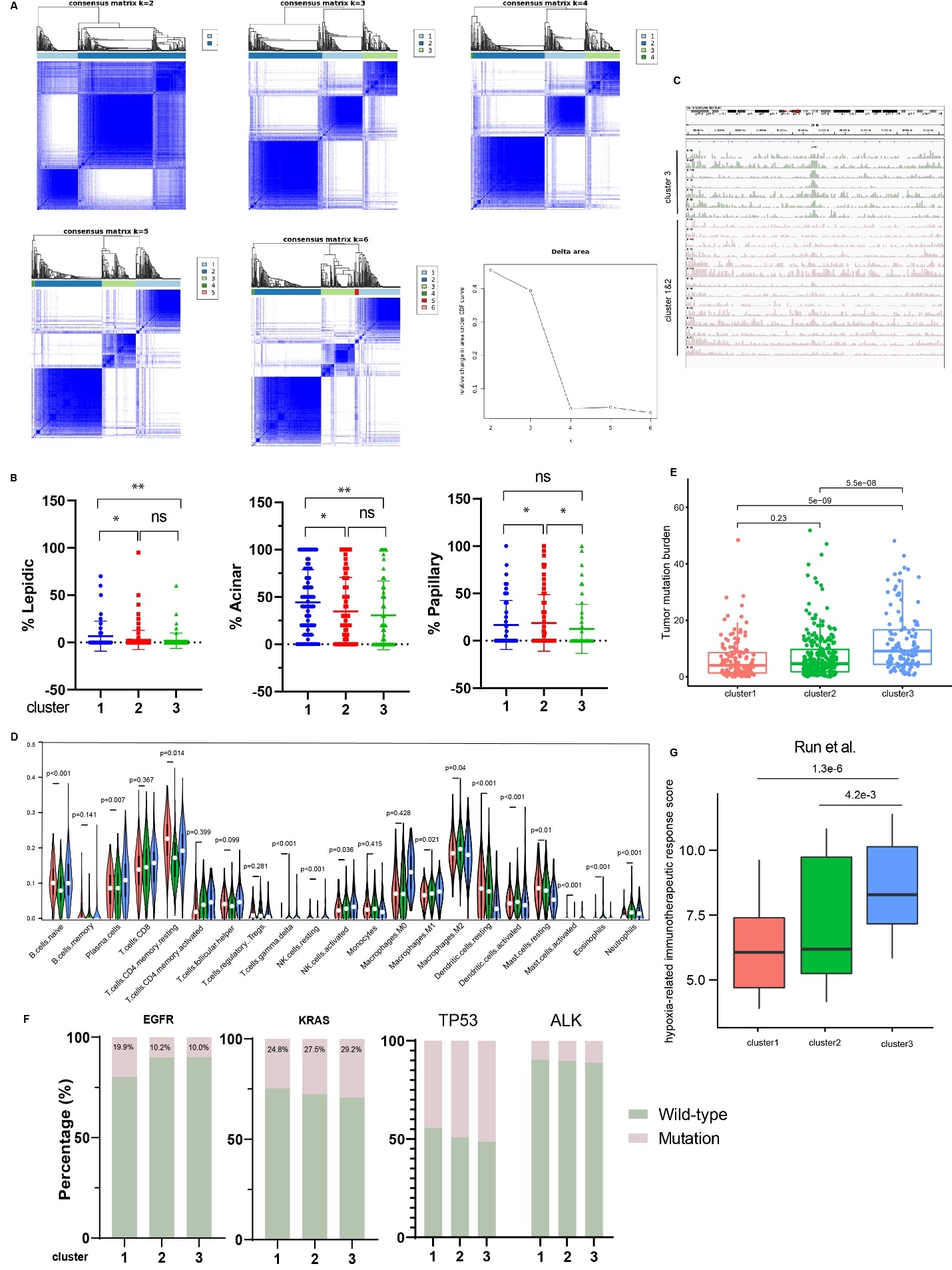


**Figure S1.** (A) Consensus matrixes of seRNA profile in TCGA-LUAD dataset for each k (k = 2–6), displaying the clustering stability using 1000 iterations of hierarchical clustering. (B) Lepidic-subtype, acinar-subtype and papillary-subtype component percentage of whole tumor tissues for the three clusters. (C) The chromatin location of specifically activated SEs in each cluster. (D) The tumor environment cell infiltration in each cluster by CIBERSORTx. (E) The tumor mutation burden of the three clusters by whole exon sequence data. (F) The percentage of samples with *EGFR*, *KRAS*, *TP53* and *ALK* mutations in each cluster. (G) The hypoxia-related immunotherapeutic response score between the three clusters. Asterisks denote statistical significance; * P<0.0.5; ** P<0.01; n.s no significance.


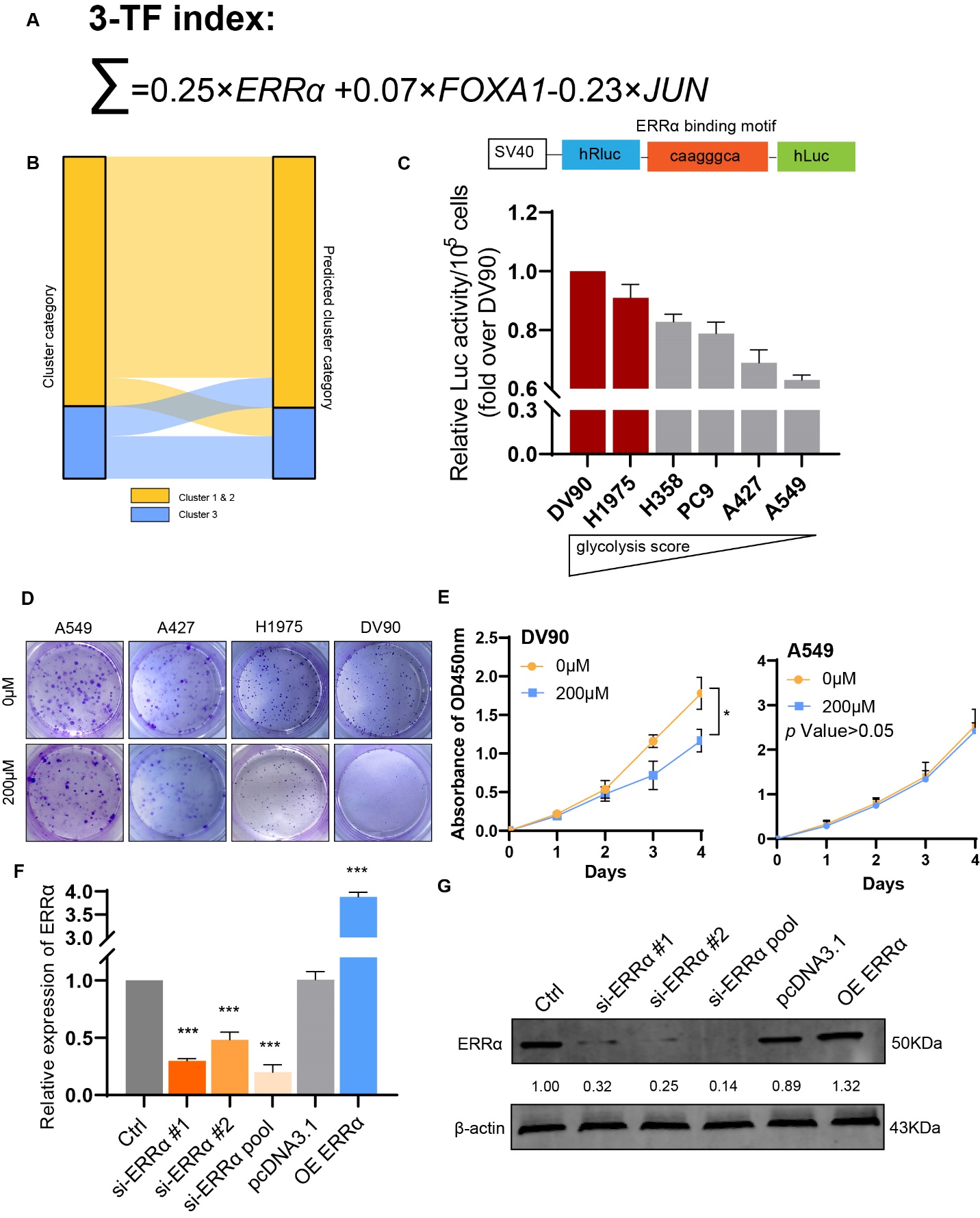


**Figure S2.** (A) The calculation formula of the 3-TF index. (B) The Sankey plot showing whether a sample is part of cluster 3 or non-cluster 3, using the clustering or the LASSO methods. (C) Transcription factor ERRα activity detection dual-luciferase reporter plasmid design and detection in 6 LUAD cell lines. Clone formation assay (D) and CCK-8 assay (E) showing that cell lines with high 3-TF index value (DV90, H1975) have higher 2-DG sensitivity than cell lines with low 3-TF index value (A549, A427). Validation of siRNA oligonucleotides and overexpression plasmids for the regulation of ERRα in mRNA (F) and protein levels (G).


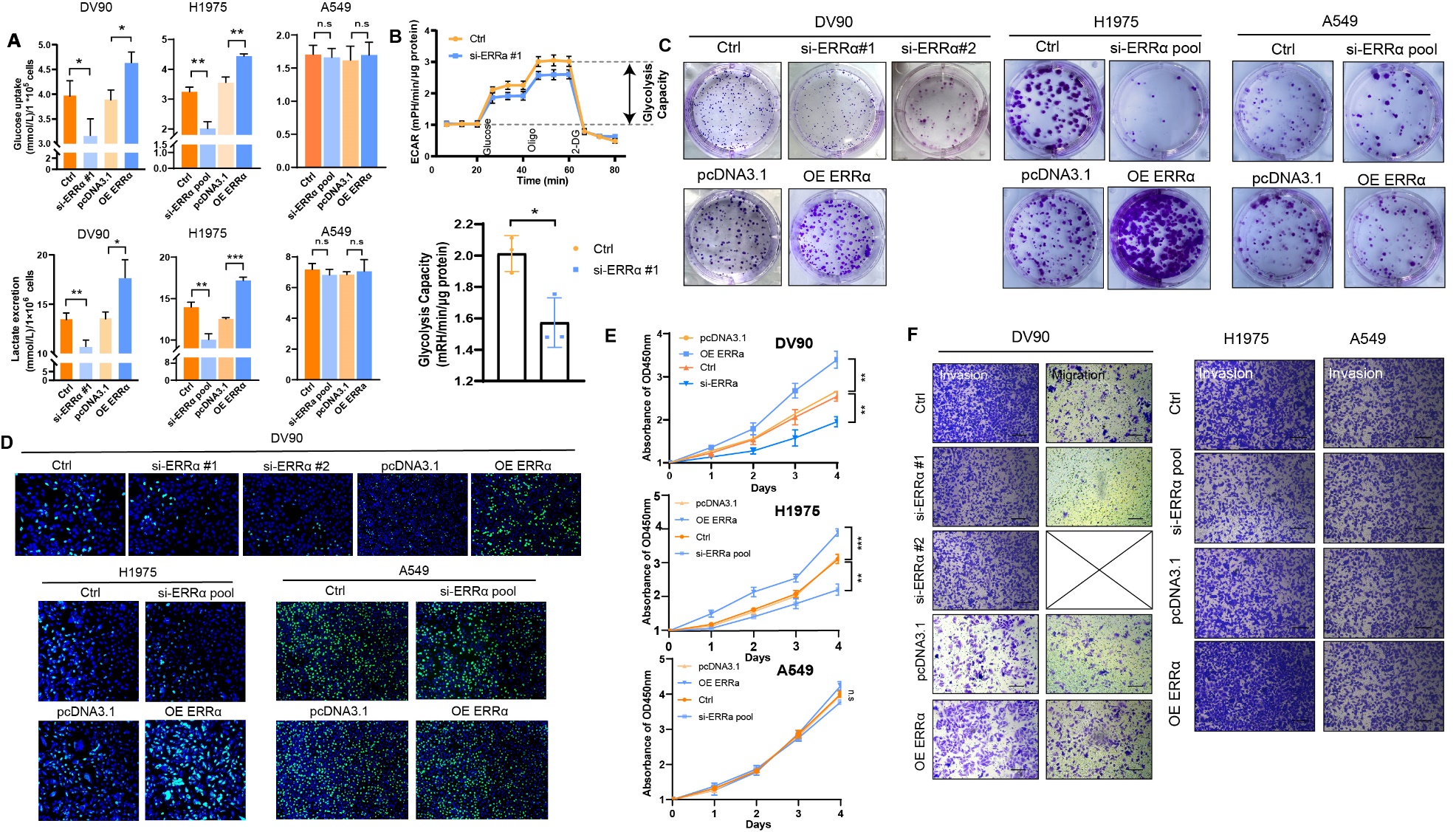


**Figure S3.** (A) Glucose uptake and lactate excretion assay based on knockdown or overexpression ERRα in DV90, H1975 and A549 cell lines. (B) Extracellular flux assays based on knockdown ERRα in DV90. Clone formation assay (C), EdU assay (D), CCK-8 cell proliferation assay (E), invasion and migration assay (F) based on knockdown or overexpression ERRα in DV90, H1975 and A549 cell lines. * P<0.0.5; ** P<0.01; *** P<0.001; n.s no significance.


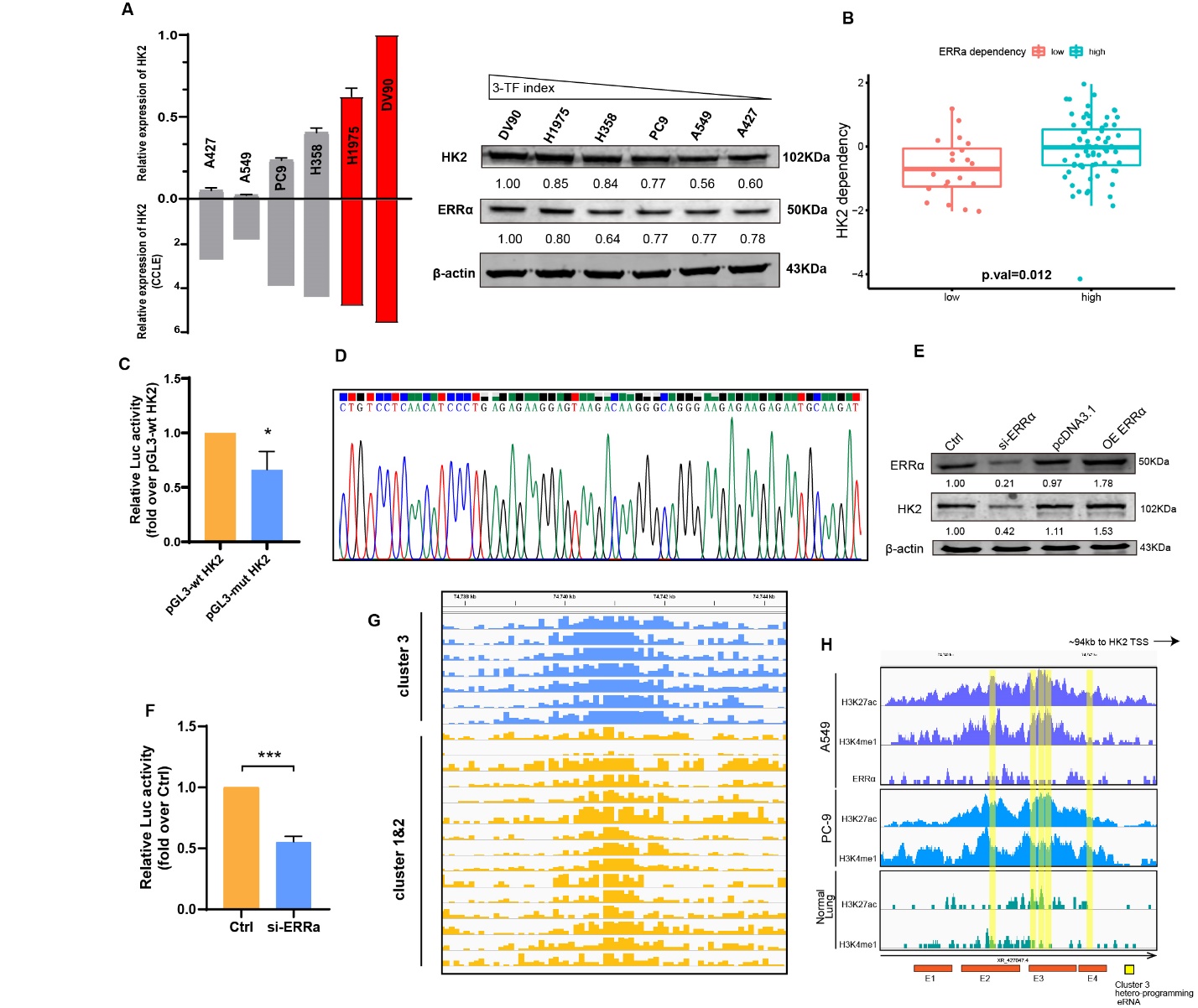


**Figure S4.** (A) The relative expression of HK2 in cell lines by qPCR analysis, RNA-seq from CCLE dataset and protein level analysis. (B) The difference in HK2 dependence between two groups based on ERRα dependency of 78 LUAD cell lines by Depmap database CRISPR screening. (C) The H1975 cells were transfected with the indicated plasmids for 48 h. The levels of luciferase activity were normalized to the pRL-TK luciferase activity. (D) The Sanger sequencing track showing the sequence of the ChIP product in the HK2 promoter region. (E) The protein expression of *HK2* regulated by ERRα. (F) The transcription activation of HK2 treated with si-ERRα or si-Control (si-Ctrl). (G) The chromatin location of the SE_XR_427047.4 locus in 22 LUAD patients with ATAC-seq in the TCGA-LUAD dataset. (H) The IGV tracks of anti-H3K27ac, anti-H3K4me1 and ERRα ChIP-seq in A549 cells, PC-9 cells or normal lung tissue from the ENCODE database.


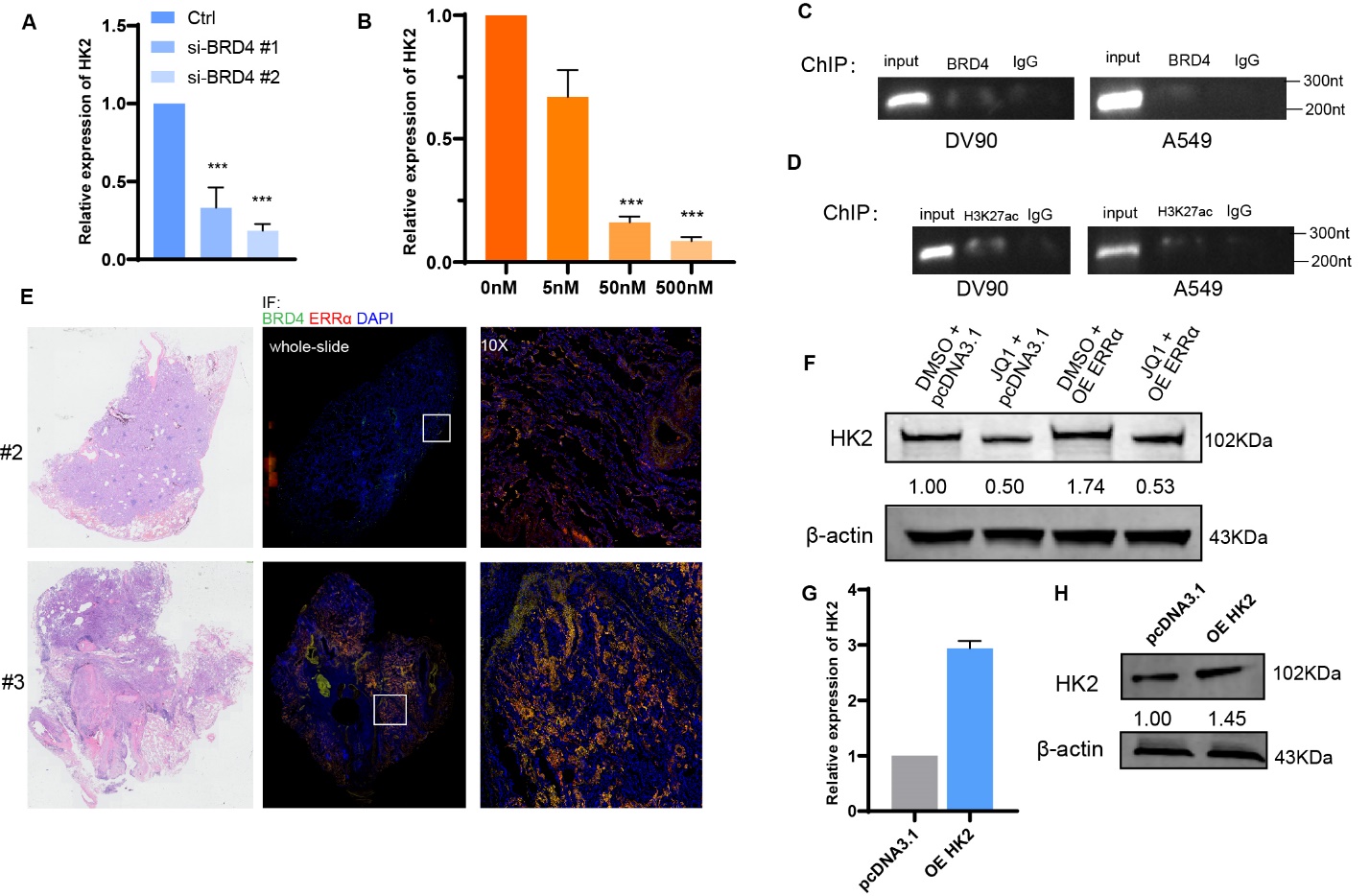


**Figure S5.** (A) The mRNA expression of HK2 regulated by interfering BRD4. (B) The mRNA expression in cells treated with different doses of JQ1. Cluster 3-like (DV90) and non-cluster 3-like (A549) cells were subjected to ChIP analysis using the anti-BRD4 antibody (C) and anti-H3K27ac antibody (D). (E) The Immunofluorescence staining for ERRα and BRD4 performed on LUAD tissue. A total of replicates were performed in slides derived from 3 LUAD patients. (F) Western-blot revealing that the ERRα regulated expression of HK2 partly depends on SEs. Validation of overexpression plasmids for the regulation of HK2 in mRNA (F) and protein levels (G).


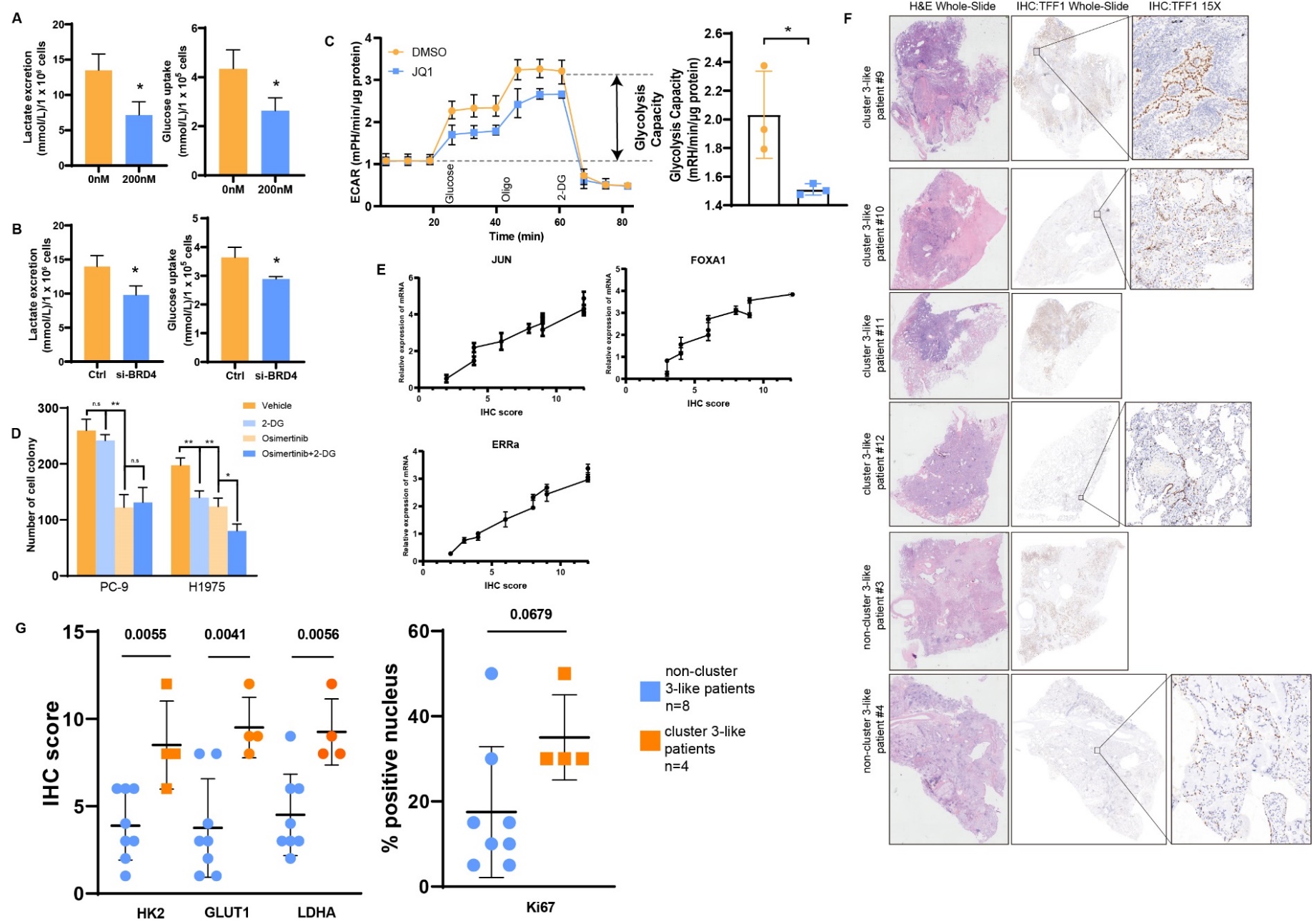


**Figure S6.** Glucose uptake and lactate excretion assays after treatment with or without JQ1 (A) and infected by siBRD4 or siCtrl (B). (C) Extracellular flux assays showing the glycolysis capacity of cells transfected with siBRD4 or si-Ctrl. (D) Clone formation assay revealing the inhibitory efficacy of 2-DG, Osimertinib and their combination in the EGFR^mut^ cluster 3-like cell line (H1975) and EGFR^mut^ non-cluster 3-like cell line (PC-9). (E) The correlation between mRNA expression and IHC score of 3 core TFs in LUAD samples from the Jiangsu Cancer Hospital. (F) Whole slide scans of H&E staining and TFF1 immunohistochemistry of patients in Fig 6B and 6C. (G) IHC scores of HK2, GLUT1 and LDHA, and percentage of ki67-positive nuclei in 12 patients with lung adenocarcinoma (including 8 non-cluster3-like patients and 4 cluster3-like patients).
