# Supplementary material for: Non-genetic stratification reveals epigenetic heterogeneity and identifies vulnerabilities of glycolysis addiction in lung adenocarcinoma subtype
Source: Oncogenesis. 2022 Oct 10;11(1):61. doi: 10.1038/s41389-022-00436-0 (PMC9550819; doi:10.1038/s41389-022-00436-0)
Supplement: Supplementary file 3 — Additional file 3 [file 41389_2022_436_MOESM3_ESM.docx]

**Title page**

**Non-genetic stratification reveals epigenetic heterogeneity and identifies vulnerabilities of glycolysis addiction in lung adenocarcinoma subtype**

**Running title:** **Epigenetic stratification shows glycolysis addiction**

**Additional file 2 Table S1-S8**

| **Table S1** The Oligonucleotides sequence: |  |
| --- | --- |
| HK2-forward | TGCCACCAGACTAAACTAGACG |
| HK2-reverse | CCCGTGCCCACAATGAGAC |
| BRD4-forward | GAGCTACCCACAGAAGAAACC |
| BRD4-reverse | GAGTCGATGCTTGAGTTGTGTT |
| SEMA4F-forward | GTGTTTGAAGTTCCCGTGGC |
| SEMA4F-reverse | CATTCACAGGCATAAGCGCC |
| TACR1-forward | TGATGTGGATCATCTTAGCCCA |
| TACR1-reverse | GAACAGGCCGTAGTACCATTC |
| M1AP-forward | CTTCCTGCCTTCAGAGTGGG |
| M1AP-reverse | GACTTGAGGAGTGGGACAGC |
| POLE4-forward | GCTTAGCAAAAGCCATGTATCCT |
| POLE4-reverse | TCTATCAAAAGGACCAGGGGC |
| actin-forward | CTCCATCCTGGCCTCGCTGT |
| actin-reverse | GCTGTCACCTTCACCGTTCC |
| HK2 promoter (co-occupied locus)-forward | GTGAGGTTAGCCAGAAACCC |
| HK2 promoter (co-occupied locus)-reverse | AAATGCACGTCCTCAACCCTC |
| SE_XR_427047.4 (long sequence)-forward | GAAACCCTCAGAGTGGAGCAC |
| SE_XR_427047.4 (long sequence)-reverse | TCCTCAACCCTCCTTCCCTAT |
| SE_XR_427047.4 E1-forward | GATCGCAGATCTCGAGGAGG |
| SE_XR_427047.4 E1-reverse | GAGGTCCACATCTTGCGGTT |
| SE_XR_427047.4 E2-forward | GATCGCAGATCTCGAGGAGG |
| SE_XR_427047.4 E2-reverse | GAGGTCCACATCTTGCGGTT |
| SE_XR_427047.4 E3-forward | TCTTGCATCCGCTTGTCTTC |
| SE_XR_427047.4 E3-reverse | GTGGCAGGATGGATGGATGAT |
| SE_XR_427047.4 E4-forward | GGGAGTAAGATGCAACTGGTG |
| SE_XR_427047.4 E4-reverse | AGCTACGTGACCACAGGTTTT |

| **Table S2** Patient ID in each cluster | |
| --- | --- |
|  | clustername |
| TCGA.05.4244_tumor | 1 |
| TCGA.05.4249_tumor | 2 |
| TCGA.05.4250_tumor | 2 |
| TCGA.05.4382_tumor | 3 |
| TCGA.05.4384_tumor | 1 |
| TCGA.05.4389_tumor | 2 |
| TCGA.05.4390_tumor | 3 |
| TCGA.05.4395_tumor | 2 |
| TCGA.05.4396_tumor | 3 |
| TCGA.05.4397_tumor | 2 |
| TCGA.05.4398_tumor | 2 |
| TCGA.05.4402_tumor | 2 |
| TCGA.05.4403_tumor | 2 |
| TCGA.05.4405_tumor | 1 |
| TCGA.05.4410_tumor | 1 |
| TCGA.05.4415_tumor | 2 |
| TCGA.05.4417_tumor | 2 |
| TCGA.05.4418_tumor | 2 |
| TCGA.05.4420_tumor | 2 |
| TCGA.05.4422_tumor | 3 |
| TCGA.05.4424_tumor | 3 |
| TCGA.05.4425_tumor | 2 |
| TCGA.05.4426_tumor | 2 |
| TCGA.05.4427_tumor | 1 |
| TCGA.05.4430_tumor | 2 |
| TCGA.05.4432_tumor | 2 |
| TCGA.05.4433_tumor | 2 |
| TCGA.05.4434_tumor | 2 |
| TCGA.05.5420_tumor | 2 |
| TCGA.05.5423_tumor | 2 |
| TCGA.05.5425_tumor | 2 |
| TCGA.05.5428_tumor | 2 |
| TCGA.05.5429_tumor | 2 |
| TCGA.05.5715_tumor | 2 |
| TCGA.35.3615_tumor | 2 |
| TCGA.35.4122_tumor | 2 |
| TCGA.35.4123_tumor | 2 |
| TCGA.35.5375_tumor | 2 |
| TCGA.38.4625_tumor | 2 |
| TCGA.38.4626_tumor | 2 |
| TCGA.38.4627_tumor | 2 |
| TCGA.38.4628_tumor | 2 |
| TCGA.38.4629_tumor | 2 |
| TCGA.38.4630_tumor | 3 |
| TCGA.38.4631_tumor | 2 |
| TCGA.38.4632_tumor | 2 |
| TCGA.38.6178_tumor | 3 |
| TCGA.38.7271_tumor | 2 |
| TCGA.38.A44F_tumor | 2 |
| TCGA.44.2655_tumor | 2 |
| TCGA.44.2656_tumor | 2 |
| TCGA.44.2657_tumor | 2 |
| TCGA.44.2659_tumor | 1 |
| TCGA.44.2661_tumor | 2 |
| TCGA.44.2662_tumor | 2 |
| TCGA.44.2665_tumor | 2 |
| TCGA.44.2666_tumor | 2 |
| TCGA.44.2668_tumor | 2 |
| TCGA.44.3396_tumor | 2 |
| TCGA.44.3398_tumor | 2 |
| TCGA.44.3917_tumor | 2 |
| TCGA.44.3918_tumor | 2 |
| TCGA.44.3919_tumor | 2 |
| TCGA.44.4112_tumor | 2 |
| TCGA.44.5643_tumor | 3 |
| TCGA.44.5644_tumor | 3 |
| TCGA.44.5645_tumor | 1 |
| TCGA.44.6145_tumor | 2 |
| TCGA.44.6146_tumor | 2 |
| TCGA.44.6147_tumor | 2 |
| TCGA.44.6148_tumor | 1 |
| TCGA.44.6774_tumor | 2 |
| TCGA.44.6775_tumor | 1 |
| TCGA.44.6776_tumor | 2 |
| TCGA.44.6777_tumor | 2 |
| TCGA.44.6778_tumor | 3 |
| TCGA.44.6779_tumor | 2 |
| TCGA.44.7659_tumor | 1 |
| TCGA.44.7660_tumor | 3 |
| TCGA.44.7661_tumor | 2 |
| TCGA.44.7662_tumor | 2 |
| TCGA.44.7667_tumor | 2 |
| TCGA.44.7669_tumor | 3 |
| TCGA.44.7670_tumor | 2 |
| TCGA.44.7671_tumor | 2 |
| TCGA.44.7672_tumor | 2 |
| TCGA.44.8117_tumor | 2 |
| TCGA.44.8119_tumor | 3 |
| TCGA.44.8120_tumor | 2 |
| TCGA.44.A479_tumor | 1 |
| TCGA.44.A47A_tumor | 1 |
| TCGA.44.A47B_tumor | 1 |
| TCGA.44.A47G_tumor | 1 |
| TCGA.44.A4SS_tumor | 3 |
| TCGA.44.A4SU_tumor | 1 |
| TCGA.49.4486_tumor | 2 |
| TCGA.49.4487_tumor | 2 |
| TCGA.49.4488_tumor | 2 |
| TCGA.49.4490_tumor | 3 |
| TCGA.49.4494_tumor | 2 |
| TCGA.49.4501_tumor | 2 |
| TCGA.49.4505_tumor | 2 |
| TCGA.49.4506_tumor | 2 |
| TCGA.49.4507_tumor | 2 |
| TCGA.49.4510_tumor | 3 |
| TCGA.49.4512_tumor | 2 |
| TCGA.49.4514_tumor | 2 |
| TCGA.49.6742_tumor | 3 |
| TCGA.49.6743_tumor | 3 |
| TCGA.49.6744_tumor | 2 |
| TCGA.49.6745_tumor | 2 |
| TCGA.49.6761_tumor | 2 |
| TCGA.49.6767_tumor | 2 |
| TCGA.49.AAQV_tumor | 1 |
| TCGA.49.AAR0_tumor | 2 |
| TCGA.49.AAR2_tumor | 2 |
| TCGA.49.AAR3_tumor | 1 |
| TCGA.49.AAR4_tumor | 1 |
| TCGA.49.AAR9_tumor | 3 |
| TCGA.49.AARE_tumor | 3 |
| TCGA.49.AARN_tumor | 1 |
| TCGA.49.AARO_tumor | 1 |
| TCGA.49.AARQ_tumor | 2 |
| TCGA.49.AARR_tumor | 1 |
| TCGA.4B.A93V_tumor | 3 |
| TCGA.50.5044_tumor | 2 |
| TCGA.50.5045_tumor | 2 |
| TCGA.50.5049_tumor | 2 |
| TCGA.50.5051_tumor | 3 |
| TCGA.50.5055_tumor | 2 |
| TCGA.50.5066_tumor | 2 |
| TCGA.50.5068_tumor | 2 |
| TCGA.50.5072_tumor | 3 |
| TCGA.50.5930_tumor | 3 |
| TCGA.50.5931_tumor | 3 |
| TCGA.50.5932_tumor | 3 |
| TCGA.50.5933_tumor | 2 |
| TCGA.50.5935_tumor | 3 |
| TCGA.50.5936_tumor | 2 |
| TCGA.50.5939_tumor | 2 |
| TCGA.50.5941_tumor | 2 |
| TCGA.50.5942_tumor | 2 |
| TCGA.50.5944_tumor | 2 |
| TCGA.50.5946_tumor | 3 |
| TCGA.50.6590_tumor | 3 |
| TCGA.50.6591_tumor | 3 |
| TCGA.50.6592_tumor | 3 |
| TCGA.50.6593_tumor | 2 |
| TCGA.50.6594_tumor | 3 |
| TCGA.50.6595_tumor | 2 |
| TCGA.50.6597_tumor | 2 |
| TCGA.50.6673_tumor | 2 |
| TCGA.50.7109_tumor | 2 |
| TCGA.50.8457_tumor | 2 |
| TCGA.50.8459_tumor | 2 |
| TCGA.50.8460_tumor | 2 |
| TCGA.53.7624_tumor | 3 |
| TCGA.53.7626_tumor | 2 |
| TCGA.53.7813_tumor | 1 |
| TCGA.53.A4EZ_tumor | 2 |
| TCGA.55.1592_tumor | 3 |
| TCGA.55.1594_tumor | 3 |
| TCGA.55.1595_tumor | 1 |
| TCGA.55.1596_tumor | 2 |
| TCGA.55.5899_tumor | 3 |
| TCGA.55.6543_tumor | 2 |
| TCGA.55.6642_tumor | 3 |
| TCGA.55.6712_tumor | 2 |
| TCGA.55.6968_tumor | 3 |
| TCGA.55.6969_tumor | 3 |
| TCGA.55.6970_tumor | 2 |
| TCGA.55.6971_tumor | 1 |
| TCGA.55.6972_tumor | 1 |
| TCGA.55.6975_tumor | 2 |
| TCGA.55.6978_tumor | 2 |
| TCGA.55.6979_tumor | 2 |
| TCGA.55.6980_tumor | 2 |
| TCGA.55.6981_tumor | 1 |
| TCGA.55.6982_tumor | 2 |
| TCGA.55.6983_tumor | 2 |
| TCGA.55.6984_tumor | 2 |
| TCGA.55.6985_tumor | 2 |
| TCGA.55.6986_tumor | 2 |
| TCGA.55.6987_tumor | 2 |
| TCGA.55.7227_tumor | 1 |
| TCGA.55.7281_tumor | 2 |
| TCGA.55.7283_tumor | 2 |
| TCGA.55.7284_tumor | 2 |
| TCGA.55.7570_tumor | 3 |
| TCGA.55.7573_tumor | 1 |
| TCGA.55.7574_tumor | 1 |
| TCGA.55.7576_tumor | 2 |
| TCGA.55.7724_tumor | 2 |
| TCGA.55.7725_tumor | 1 |
| TCGA.55.7726_tumor | 2 |
| TCGA.55.7727_tumor | 1 |
| TCGA.55.7728_tumor | 2 |
| TCGA.55.7815_tumor | 2 |
| TCGA.55.7816_tumor | 2 |
| TCGA.55.7903_tumor | 1 |
| TCGA.55.7907_tumor | 3 |
| TCGA.55.7910_tumor | 3 |
| TCGA.55.7911_tumor | 1 |
| TCGA.55.7913_tumor | 3 |
| TCGA.55.7914_tumor | 1 |
| TCGA.55.7994_tumor | 2 |
| TCGA.55.7995_tumor | 1 |
| TCGA.55.8085_tumor | 3 |
| TCGA.55.8087_tumor | 1 |
| TCGA.55.8089_tumor | 2 |
| TCGA.55.8090_tumor | 2 |
| TCGA.55.8091_tumor | 2 |
| TCGA.55.8092_tumor | 3 |
| TCGA.55.8094_tumor | 3 |
| TCGA.55.8096_tumor | 1 |
| TCGA.55.8097_tumor | 1 |
| TCGA.55.8203_tumor | 2 |
| TCGA.55.8204_tumor | 2 |
| TCGA.55.8205_tumor | 2 |
| TCGA.55.8206_tumor | 1 |
| TCGA.55.8207_tumor | 1 |
| TCGA.55.8208_tumor | 2 |
| TCGA.55.8299_tumor | 2 |
| TCGA.55.8301_tumor | 2 |
| TCGA.55.8302_tumor | 1 |
| TCGA.55.8505_tumor | 3 |
| TCGA.55.8506_tumor | 2 |
| TCGA.55.8507_tumor | 3 |
| TCGA.55.8508_tumor | 3 |
| TCGA.55.8510_tumor | 1 |
| TCGA.55.8511_tumor | 1 |
| TCGA.55.8512_tumor | 1 |
| TCGA.55.8513_tumor | 1 |
| TCGA.55.8514_tumor | 1 |
| TCGA.55.8614_tumor | 3 |
| TCGA.55.8615_tumor | 3 |
| TCGA.55.8616_tumor | 2 |
| TCGA.55.8619_tumor | 2 |
| TCGA.55.8620_tumor | 3 |
| TCGA.55.8621_tumor | 1 |
| TCGA.55.A48X_tumor | 1 |
| TCGA.55.A48Y_tumor | 2 |
| TCGA.55.A48Z_tumor | 1 |
| TCGA.55.A490_tumor | 3 |
| TCGA.55.A491_tumor | 1 |
| TCGA.55.A492_tumor | 2 |
| TCGA.55.A493_tumor | 1 |
| TCGA.55.A494_tumor | 3 |
| TCGA.55.A4DF_tumor | 1 |
| TCGA.55.A4DG_tumor | 1 |
| TCGA.55.A57B_tumor | 1 |
| TCGA.62.8394_tumor | 3 |
| TCGA.62.8395_tumor | 1 |
| TCGA.62.8397_tumor | 2 |
| TCGA.62.8398_tumor | 3 |
| TCGA.62.8399_tumor | 3 |
| TCGA.62.8402_tumor | 3 |
| TCGA.62.A46O_tumor | 3 |
| TCGA.62.A46P_tumor | 2 |
| TCGA.62.A46R_tumor | 1 |
| TCGA.62.A46S_tumor | 1 |
| TCGA.62.A46U_tumor | 1 |
| TCGA.62.A46V_tumor | 1 |
| TCGA.62.A46Y_tumor | 1 |
| TCGA.62.A470_tumor | 1 |
| TCGA.62.A471_tumor | 3 |
| TCGA.62.A472_tumor | 1 |
| TCGA.64.1676_tumor | 2 |
| TCGA.64.1677_tumor | 2 |
| TCGA.64.1678_tumor | 2 |
| TCGA.64.1679_tumor | 2 |
| TCGA.64.1680_tumor | 2 |
| TCGA.64.1681_tumor | 2 |
| TCGA.64.5774_tumor | 3 |
| TCGA.64.5775_tumor | 2 |
| TCGA.64.5778_tumor | 2 |
| TCGA.64.5779_tumor | 1 |
| TCGA.64.5781_tumor | 2 |
| TCGA.64.5815_tumor | 2 |
| TCGA.67.3770_tumor | 2 |
| TCGA.67.3771_tumor | 3 |
| TCGA.67.3772_tumor | 2 |
| TCGA.67.3773_tumor | 2 |
| TCGA.67.3774_tumor | 2 |
| TCGA.67.4679_tumor | 1 |
| TCGA.67.6215_tumor | 2 |
| TCGA.67.6216_tumor | 2 |
| TCGA.67.6217_tumor | 1 |
| TCGA.69.7760_tumor | 3 |
| TCGA.69.7761_tumor | 3 |
| TCGA.69.7763_tumor | 2 |
| TCGA.69.7764_tumor | 1 |
| TCGA.69.7765_tumor | 1 |
| TCGA.69.7973_tumor | 3 |
| TCGA.69.7974_tumor | 2 |
| TCGA.69.7978_tumor | 2 |
| TCGA.69.7979_tumor | 2 |
| TCGA.69.7980_tumor | 3 |
| TCGA.69.8253_tumor | 2 |
| TCGA.69.8254_tumor | 2 |
| TCGA.69.8255_tumor | 2 |
| TCGA.69.8453_tumor | 2 |
| TCGA.69.A59K_tumor | 1 |
| TCGA.71.6725_tumor | 2 |
| TCGA.71.8520_tumor | 1 |
| TCGA.73.4658_tumor | 2 |
| TCGA.73.4659_tumor | 3 |
| TCGA.73.4662_tumor | 1 |
| TCGA.73.4666_tumor | 2 |
| TCGA.73.4668_tumor | 2 |
| TCGA.73.4670_tumor | 2 |
| TCGA.73.4675_tumor | 2 |
| TCGA.73.4676_tumor | 2 |
| TCGA.73.4677_tumor | 2 |
| TCGA.73.7498_tumor | 1 |
| TCGA.73.7499_tumor | 2 |
| TCGA.73.A9RS_tumor | 3 |
| TCGA.75.5122_tumor | 2 |
| TCGA.75.5125_tumor | 2 |
| TCGA.75.5126_tumor | 2 |
| TCGA.75.5146_tumor | 2 |
| TCGA.75.5147_tumor | 2 |
| TCGA.75.6203_tumor | 2 |
| TCGA.75.6205_tumor | 2 |
| TCGA.75.6206_tumor | 1 |
| TCGA.75.6207_tumor | 2 |
| TCGA.75.6211_tumor | 3 |
| TCGA.75.6212_tumor | 2 |
| TCGA.75.6214_tumor | 2 |
| TCGA.75.7025_tumor | 1 |
| TCGA.75.7027_tumor | 3 |
| TCGA.75.7030_tumor | 2 |
| TCGA.75.7031_tumor | 3 |
| TCGA.78.7143_tumor | 2 |
| TCGA.78.7145_tumor | 2 |
| TCGA.78.7146_tumor | 3 |
| TCGA.78.7147_tumor | 2 |
| TCGA.78.7148_tumor | 2 |
| TCGA.78.7149_tumor | 2 |
| TCGA.78.7150_tumor | 3 |
| TCGA.78.7152_tumor | 2 |
| TCGA.78.7153_tumor | 2 |
| TCGA.78.7154_tumor | 2 |
| TCGA.78.7155_tumor | 3 |
| TCGA.78.7156_tumor | 1 |
| TCGA.78.7158_tumor | 1 |
| TCGA.78.7159_tumor | 3 |
| TCGA.78.7160_tumor | 2 |
| TCGA.78.7161_tumor | 3 |
| TCGA.78.7162_tumor | 2 |
| TCGA.78.7163_tumor | 2 |
| TCGA.78.7166_tumor | 2 |
| TCGA.78.7167_tumor | 3 |
| TCGA.78.7220_tumor | 3 |
| TCGA.78.7535_tumor | 2 |
| TCGA.78.7536_tumor | 3 |
| TCGA.78.7537_tumor | 2 |
| TCGA.78.7539_tumor | 3 |
| TCGA.78.7540_tumor | 2 |
| TCGA.78.7542_tumor | 2 |
| TCGA.78.7633_tumor | 2 |
| TCGA.78.8640_tumor | 3 |
| TCGA.78.8648_tumor | 2 |
| TCGA.78.8655_tumor | 2 |
| TCGA.78.8660_tumor | 3 |
| TCGA.78.8662_tumor | 3 |
| TCGA.80.5607_tumor | 2 |
| TCGA.80.5608_tumor | 3 |
| TCGA.80.5611_tumor | 3 |
| TCGA.83.5908_tumor | 1 |
| TCGA.86.6562_tumor | 2 |
| TCGA.86.6851_tumor | 1 |
| TCGA.86.7701_tumor | 1 |
| TCGA.86.7711_tumor | 2 |
| TCGA.86.7713_tumor | 3 |
| TCGA.86.7714_tumor | 1 |
| TCGA.86.7953_tumor | 1 |
| TCGA.86.7954_tumor | 3 |
| TCGA.86.7955_tumor | 3 |
| TCGA.86.8054_tumor | 3 |
| TCGA.86.8055_tumor | 2 |
| TCGA.86.8056_tumor | 2 |
| TCGA.86.8073_tumor | 2 |
| TCGA.86.8074_tumor | 2 |
| TCGA.86.8075_tumor | 1 |
| TCGA.86.8076_tumor | 2 |
| TCGA.86.8278_tumor | 2 |
| TCGA.86.8279_tumor | 1 |
| TCGA.86.8280_tumor | 2 |
| TCGA.86.8281_tumor | 1 |
| TCGA.86.8358_tumor | 3 |
| TCGA.86.8359_tumor | 3 |
| TCGA.86.8585_tumor | 3 |
| TCGA.86.8668_tumor | 2 |
| TCGA.86.8669_tumor | 3 |
| TCGA.86.8671_tumor | 1 |
| TCGA.86.8672_tumor | 2 |
| TCGA.86.8673_tumor | 2 |
| TCGA.86.8674_tumor | 2 |
| TCGA.86.A456_tumor | 1 |
| TCGA.86.A4D0_tumor | 3 |
| TCGA.86.A4JF_tumor | 2 |
| TCGA.86.A4P7_tumor | 1 |
| TCGA.86.A4P8_tumor | 1 |
| TCGA.91.6828_tumor | 1 |
| TCGA.91.6829_tumor | 3 |
| TCGA.91.6830_tumor | 3 |
| TCGA.91.6831_tumor | 3 |
| TCGA.91.6835_tumor | 1 |
| TCGA.91.6836_tumor | 3 |
| TCGA.91.6840_tumor | 3 |
| TCGA.91.6847_tumor | 3 |
| TCGA.91.6848_tumor | 2 |
| TCGA.91.6849_tumor | 2 |
| TCGA.91.7771_tumor | 1 |
| TCGA.91.8496_tumor | 2 |
| TCGA.91.8497_tumor | 1 |
| TCGA.91.8499_tumor | 3 |
| TCGA.91.A4BC_tumor | 3 |
| TCGA.91.A4BD_tumor | 2 |
| TCGA.93.7347_tumor | 2 |
| TCGA.93.7348_tumor | 2 |
| TCGA.93.8067_tumor | 2 |
| TCGA.93.A4JN_tumor | 1 |
| TCGA.93.A4JO_tumor | 1 |
| TCGA.93.A4JP_tumor | 1 |
| TCGA.93.A4JQ_tumor | 1 |
| TCGA.95.7039_tumor | 2 |
| TCGA.95.7043_tumor | 3 |
| TCGA.95.7562_tumor | 2 |
| TCGA.95.7567_tumor | 2 |
| TCGA.95.7944_tumor | 2 |
| TCGA.95.7947_tumor | 1 |
| TCGA.95.7948_tumor | 2 |
| TCGA.95.8039_tumor | 2 |
| TCGA.95.8494_tumor | 1 |
| TCGA.95.A4VK_tumor | 1 |
| TCGA.95.A4VN_tumor | 1 |
| TCGA.95.A4VP_tumor | 2 |
| TCGA.97.7546_tumor | 2 |
| TCGA.97.7547_tumor | 2 |
| TCGA.97.7552_tumor | 2 |
| TCGA.97.7553_tumor | 2 |
| TCGA.97.7554_tumor | 2 |
| TCGA.97.7937_tumor | 1 |
| TCGA.97.7938_tumor | 1 |
| TCGA.97.7941_tumor | 2 |
| TCGA.97.8171_tumor | 1 |
| TCGA.97.8172_tumor | 1 |
| TCGA.97.8174_tumor | 1 |
| TCGA.97.8175_tumor | 2 |
| TCGA.97.8176_tumor | 3 |
| TCGA.97.8177_tumor | 2 |
| TCGA.97.8179_tumor | 1 |
| TCGA.97.8547_tumor | 1 |
| TCGA.97.8552_tumor | 1 |
| TCGA.97.A4LX_tumor | 1 |
| TCGA.97.A4M0_tumor | 1 |
| TCGA.97.A4M1_tumor | 1 |
| TCGA.97.A4M2_tumor | 1 |
| TCGA.97.A4M3_tumor | 1 |
| TCGA.97.A4M5_tumor | 1 |
| TCGA.97.A4M6_tumor | 1 |
| TCGA.97.A4M7_tumor | 1 |
| TCGA.99.7458_tumor | 1 |
| TCGA.99.8025_tumor | 2 |
| TCGA.99.8028_tumor | 2 |
| TCGA.99.8032_tumor | 3 |
| TCGA.99.8033_tumor | 2 |
| TCGA.99.AA5R_tumor | 1 |
| TCGA.J2.8192_tumor | 2 |
| TCGA.J2.8194_tumor | 2 |
| TCGA.J2.A4AD_tumor | 3 |
| TCGA.J2.A4AE_tumor | 1 |
| TCGA.J2.A4AG_tumor | 1 |
| TCGA.L4.A4E5_tumor | 1 |
| TCGA.L4.A4E6_tumor | 1 |
| TCGA.L9.A443_tumor | 2 |
| TCGA.L9.A444_tumor | 1 |
| TCGA.L9.A50W_tumor | 1 |
| TCGA.L9.A5IP_tumor | 3 |
| TCGA.L9.A743_tumor | 1 |
| TCGA.L9.A7SV_tumor | 1 |
| TCGA.L9.A8F4_tumor | 1 |
| TCGA.MN.A4N1_tumor | 2 |
| TCGA.MN.A4N4_tumor | 3 |
| TCGA.MN.A4N5_tumor | 1 |
| TCGA.MP.A4SV_tumor | 1 |
| TCGA.MP.A4SW_tumor | 1 |
| TCGA.MP.A4SY_tumor | 1 |
| TCGA.MP.A4T4_tumor | 1 |
| TCGA.MP.A4T6_tumor | 1 |
| TCGA.MP.A4T7_tumor | 3 |
| TCGA.MP.A4T8_tumor | 3 |
| TCGA.MP.A4T9_tumor | 1 |
| TCGA.MP.A4TA_tumor | 3 |
| TCGA.MP.A4TC_tumor | 2 |
| TCGA.MP.A4TD_tumor | 3 |
| TCGA.MP.A4TE_tumor | 3 |
| TCGA.MP.A4TF_tumor | 3 |
| TCGA.MP.A4TH_tumor | 1 |
| TCGA.MP.A4TI_tumor | 1 |
| TCGA.MP.A4TJ_tumor | 1 |
| TCGA.MP.A4TK_tumor | 1 |
| TCGA.MP.A5C7_tumor | 1 |
| TCGA.NJ.A4YF_tumor | 3 |
| TCGA.NJ.A4YG_tumor | 2 |
| TCGA.NJ.A4YI_tumor | 3 |
| TCGA.NJ.A4YP_tumor | 2 |
| TCGA.NJ.A4YQ_tumor | 3 |
| TCGA.NJ.A55A_tumor | 1 |
| TCGA.NJ.A55O_tumor | 3 |
| TCGA.NJ.A55R_tumor | 3 |
| TCGA.NJ.A7XG_tumor | 1 |
| TCGA.O1.A52J_tumor | 3 |
| TCGA.S2.AA1A_tumor | 1 |

| **Table S3** The chromatin location of specifically activated SEs in each cluster. | | | | | |
| --- | --- | --- | --- | --- | --- |
|  | chr | star | stop | logFC | clustername |
| chr1:53381774 | chr1 | 53381774 | 53381874 | 1.835935 | eRNAcluster1 |
| chr1:53381734 | chr1 | 53381734 | 53381834 | 1.774649 | eRNAcluster1 |
| chr1:53383104 | chr1 | 53383104 | 53383204 | 1.850951 | eRNAcluster1 |
| chr1:53381114 | chr1 | 53381114 | 53381214 | 1.8238 | eRNAcluster1 |
| chr1:53383467 | chr1 | 53383467 | 53383567 | 1.707379 | eRNAcluster1 |
| chr1:53381624 | chr1 | 53381624 | 53381724 | 1.779049 | eRNAcluster1 |
| chr1:53382344 | chr1 | 53382344 | 53382444 | 1.758774 | eRNAcluster1 |
| chr1:53382464 | chr1 | 53382464 | 53382564 | 1.764967 | eRNAcluster1 |
| chr1:53381084 | chr1 | 53381084 | 53381184 | 1.78203 | eRNAcluster1 |
| chr1:35647337 | chr1 | 35647337 | 35647437 | 1.594867 | eRNAcluster1 |
| chr1:53382004 | chr1 | 53382004 | 53382104 | 1.809206 | eRNAcluster1 |
| chr1:53382124 | chr1 | 53382124 | 53382224 | 1.730359 | eRNAcluster1 |
| chr1:35650604 | chr1 | 35650604 | 35650704 | 1.585481 | eRNAcluster1 |
| chr1:53381594 | chr1 | 53381594 | 53381694 | 1.766711 | eRNAcluster1 |
| chr1:53381664 | chr1 | 53381664 | 53381764 | 1.72804 | eRNAcluster1 |
| chr1:35651644 | chr1 | 35651644 | 35651744 | 1.607168 | eRNAcluster1 |
| chr1:53382864 | chr1 | 53382864 | 53382964 | 1.637494 | eRNAcluster1 |
| chr1:35647557 | chr1 | 35647557 | 35647657 | 1.632708 | eRNAcluster1 |
| chr1:53382834 | chr1 | 53382834 | 53382934 | 1.630451 | eRNAcluster1 |
| chr1:53383797 | chr1 | 53383797 | 53383897 | 1.550079 | eRNAcluster1 |
| chr1:53381394 | chr1 | 53381394 | 53381494 | 1.727252 | eRNAcluster1 |
| chr1:35651854 | chr1 | 35651854 | 35651954 | 1.628148 | eRNAcluster1 |
| chr1:53385937 | chr1 | 53385937 | 53386037 | 1.615332 | eRNAcluster1 |
| chr1:35647527 | chr1 | 35647527 | 35647627 | 1.584459 | eRNAcluster1 |
| chr1:53383707 | chr1 | 53383707 | 53383807 | 1.611676 | eRNAcluster1 |
| chr1:35651884 | chr1 | 35651884 | 35651984 | 1.608811 | eRNAcluster1 |
| chr1:53384547 | chr1 | 53384547 | 53384647 | 1.597219 | eRNAcluster1 |
| chr1:35651014 | chr1 | 35651014 | 35651114 | 1.618625 | eRNAcluster1 |
| chr21:34943114 | chr21 | 34943114 | 34943214 | 1.524599 | eRNAcluster1 |
| chr1:53383757 | chr1 | 53383757 | 53383857 | 1.542504 | eRNAcluster1 |
| chr1:35651824 | chr1 | 35651824 | 35651924 | 1.555365 | eRNAcluster1 |
| chr21:34943084 | chr21 | 34943084 | 34943184 | 1.534755 | eRNAcluster1 |
| chr6:76614448 | chr6 | 76614448 | 76614548 | 1.596989 | eRNAcluster1 |
| chr1:35647697 | chr1 | 35647697 | 35647797 | 1.515502 | eRNAcluster1 |
| chr1:35651614 | chr1 | 35651614 | 35651714 | 1.639062 | eRNAcluster1 |
| chr1:35652054 | chr1 | 35652054 | 35652154 | 1.645776 | eRNAcluster1 |
| chr1:35651454 | chr1 | 35651454 | 35651554 | 1.595945 | eRNAcluster1 |
| chr21:34943714 | chr21 | 34943714 | 34943814 | 1.632431 | eRNAcluster1 |
| chr1:35647607 | chr1 | 35647607 | 35647707 | 1.520209 | eRNAcluster1 |
| chr1:35650684 | chr1 | 35650684 | 35650784 | 1.586169 | eRNAcluster1 |
| chr1:35651424 | chr1 | 35651424 | 35651524 | 1.600018 | eRNAcluster1 |
| chr1:234544641 | chr1 | 2.35E+08 | 2.35E+08 | 1.639782 | eRNAcluster1 |
| chr1:53384147 | chr1 | 53384147 | 53384247 | 1.526949 | eRNAcluster1 |
| chr17:61876696 | chr17 | 61876696 | 61876796 | 1.571245 | eRNAcluster1 |
| chr1:245010445 | chr1 | 2.45E+08 | 2.45E+08 | 1.573321 | eRNAcluster1 |
| chr1:234545161 | chr1 | 2.35E+08 | 2.35E+08 | 1.6226 | eRNAcluster1 |
| chr6:76616878 | chr6 | 76616878 | 76616978 | 1.686361 | eRNAcluster1 |
| chr1:234544011 | chr1 | 2.35E+08 | 2.35E+08 | 1.532376 | eRNAcluster1 |
| chr1:35652364 | chr1 | 35652364 | 35652464 | 1.61376 | eRNAcluster1 |
| chr1:35651914 | chr1 | 35651914 | 35652014 | 1.540653 | eRNAcluster1 |
| chr1:35651384 | chr1 | 35651384 | 35651484 | 1.580623 | eRNAcluster1 |
| chr1:234543881 | chr1 | 2.35E+08 | 2.35E+08 | 1.551008 | eRNAcluster1 |
| chr1:234544701 | chr1 | 2.35E+08 | 2.35E+08 | 1.564033 | eRNAcluster1 |
| chr1:234545801 | chr1 | 2.35E+08 | 2.35E+08 | 1.538371 | eRNAcluster1 |
| chr17:61876656 | chr17 | 61876656 | 61876756 | 1.504624 | eRNAcluster1 |
| chr21:34943744 | chr21 | 34943744 | 34943844 | 1.716861 | eRNAcluster1 |
| chr17:61876276 | chr17 | 61876276 | 61876376 | 1.557323 | eRNAcluster1 |
| chr21:34942834 | chr21 | 34942834 | 34942934 | 1.55123 | eRNAcluster1 |
| chr6:76614298 | chr6 | 76614298 | 76614398 | 1.50624 | eRNAcluster1 |
| chr1:53383927 | chr1 | 53383927 | 53384027 | 1.529657 | eRNAcluster1 |
| chr1:234544101 | chr1 | 2.35E+08 | 2.35E+08 | 1.567866 | eRNAcluster1 |
| chr21:34943144 | chr21 | 34943144 | 34943244 | 1.505969 | eRNAcluster1 |
| chr21:34943554 | chr21 | 34943554 | 34943654 | 1.637805 | eRNAcluster1 |
| chr1:234544341 | chr1 | 2.35E+08 | 2.35E+08 | 1.534975 | eRNAcluster1 |
| chr17:73592117 | chr17 | 73592117 | 73592217 | 1.556397 | eRNAcluster1 |
| chr21:34943674 | chr21 | 34943674 | 34943774 | 1.551902 | eRNAcluster1 |
| chr14:105854794 | chr14 | 1.06E+08 | 1.06E+08 | 1.51444 | eRNAcluster1 |
| chr1:53384847 | chr1 | 53384847 | 53384947 | 1.508036 | eRNAcluster1 |
| chr17:61876216 | chr17 | 61876216 | 61876316 | 1.536737 | eRNAcluster1 |
| chr17:73592087 | chr17 | 73592087 | 73592187 | 1.557213 | eRNAcluster1 |
| chr21:34942754 | chr21 | 34942754 | 34942854 | 1.508597 | eRNAcluster1 |
| chr6:76616558 | chr6 | 76616558 | 76616658 | 1.547107 | eRNAcluster1 |
| chr1:234543821 | chr1 | 2.35E+08 | 2.35E+08 | 1.570405 | eRNAcluster1 |
| chr17:61876246 | chr17 | 61876246 | 61876346 | 1.500837 | eRNAcluster1 |
| chr16:11026299 | chr16 | 11026299 | 11026399 | 1.558891 | eRNAcluster1 |
| chr1:234544061 | chr1 | 2.35E+08 | 2.35E+08 | 1.525661 | eRNAcluster1 |
| chr6:76604230 | chr6 | 76604230 | 76604330 | 1.556925 | eRNAcluster1 |
| chr17:73592057 | chr17 | 73592057 | 73592157 | 1.563344 | eRNAcluster1 |
| chr1:245010605 | chr1 | 2.45E+08 | 2.45E+08 | 1.505288 | eRNAcluster1 |
| chr6:76604200 | chr6 | 76604200 | 76604300 | 1.568872 | eRNAcluster1 |
| chr16:11027469 | chr16 | 11027469 | 11027569 | 1.552869 | eRNAcluster1 |
| chr17:73591737 | chr17 | 73591737 | 73591837 | 1.602852 | eRNAcluster1 |
| chr17:73592337 | chr17 | 73592337 | 73592437 | 1.507153 | eRNAcluster1 |
| chr17:73591447 | chr17 | 73591447 | 73591547 | 1.502634 | eRNAcluster1 |
| chr16:11029331 | chr16 | 11029331 | 11029431 | 1.593814 | eRNAcluster1 |
| chr16:11029581 | chr16 | 11029581 | 11029681 | 1.569056 | eRNAcluster1 |
| chr16:11024224 | chr16 | 11024224 | 11024324 | 1.535833 | eRNAcluster1 |
| chr16:11027981 | chr16 | 11027981 | 11028081 | 1.536781 | eRNAcluster1 |
| chr16:11024554 | chr16 | 11024554 | 11024654 | 1.512158 | eRNAcluster1 |
| chr13:113531328 | chr13 | 1.14E+08 | 1.14E+08 | 1.635342 | eRNAcluster1 |
| chr16:11024614 | chr16 | 11024614 | 11024714 | 1.532486 | eRNAcluster1 |
| chr13:113531408 | chr13 | 1.14E+08 | 1.14E+08 | 1.636757 | eRNAcluster1 |
| chr16:11025314 | chr16 | 11025314 | 11025414 | 1.540369 | eRNAcluster1 |
| chr16:11025544 | chr16 | 11025544 | 11025644 | 1.50928 | eRNAcluster1 |
| chr13:113534303 | chr13 | 1.14E+08 | 1.14E+08 | 1.678433 | eRNAcluster1 |
| chr16:11024644 | chr16 | 11024644 | 11024744 | 1.516902 | eRNAcluster1 |
| chr16:11025234 | chr16 | 11025234 | 11025334 | 1.50886 | eRNAcluster1 |
| chr13:113530738 | chr13 | 1.14E+08 | 1.14E+08 | 1.53547 | eRNAcluster1 |
| chr13:113530778 | chr13 | 1.14E+08 | 1.14E+08 | 1.55829 | eRNAcluster1 |
| chr13:113530838 | chr13 | 1.14E+08 | 1.14E+08 | 1.538615 | eRNAcluster1 |
| chr2:160959135 | chr2 | 1.61E+08 | 1.61E+08 | 1.560077 | eRNAcluster1 |
| chr13:113534343 | chr13 | 1.14E+08 | 1.14E+08 | 1.716862 | eRNAcluster1 |
| chr13:113534513 | chr13 | 1.14E+08 | 1.14E+08 | 1.638203 | eRNAcluster1 |
| chr13:113534253 | chr13 | 1.14E+08 | 1.14E+08 | 1.644785 | eRNAcluster1 |
| chr6:25107708 | chr6 | 25107708 | 25107808 | 1.506919 | eRNAcluster1 |
| chr13:113533963 | chr13 | 1.14E+08 | 1.14E+08 | 1.51839 | eRNAcluster1 |
| chr13:113534053 | chr13 | 1.14E+08 | 1.14E+08 | 1.535412 | eRNAcluster1 |
| chr13:113533993 | chr13 | 1.14E+08 | 1.14E+08 | 1.531596 | eRNAcluster1 |
| chr3:193119392 | chr3 | 1.93E+08 | 1.93E+08 | 1.809054 | eRNAcluster1 |
| chr3:193119692 | chr3 | 1.93E+08 | 1.93E+08 | 1.812158 | eRNAcluster1 |
| chr3:193118632 | chr3 | 1.93E+08 | 1.93E+08 | 1.846456 | eRNAcluster1 |
| chr3:193119752 | chr3 | 1.93E+08 | 1.93E+08 | 1.701914 | eRNAcluster1 |
| chr3:193117902 | chr3 | 1.93E+08 | 1.93E+08 | 1.860762 | eRNAcluster1 |
| chr3:193117802 | chr3 | 1.93E+08 | 1.93E+08 | 1.82062 | eRNAcluster1 |
| chr3:193118332 | chr3 | 1.93E+08 | 1.93E+08 | 1.833341 | eRNAcluster1 |
| chr3:193117052 | chr3 | 1.93E+08 | 1.93E+08 | 1.884487 | eRNAcluster1 |
| chr3:193117232 | chr3 | 1.93E+08 | 1.93E+08 | 1.850902 | eRNAcluster1 |
| chr3:193117402 | chr3 | 1.93E+08 | 1.93E+08 | 1.815388 | eRNAcluster1 |
| chr3:193117292 | chr3 | 1.93E+08 | 1.93E+08 | 1.816082 | eRNAcluster1 |
| chr3:193117592 | chr3 | 1.93E+08 | 1.93E+08 | 1.734932 | eRNAcluster1 |
| chr3:193116952 | chr3 | 1.93E+08 | 1.93E+08 | 1.826609 | eRNAcluster1 |
| chrX:47054103 | chrX | 47054103 | 47054203 | -1.45805 | eRNAcluster2 |
| chr1:356473371 | chr1 | 35647337 | 35647437 | -1.42491 | eRNAcluster2 |
| chr1:356476971 | chr1 | 35647697 | 35647797 | -1.42281 | eRNAcluster2 |
| chr6:76613578 | chr6 | 76613578 | 76613678 | -1.46665 | eRNAcluster2 |
| chr1:356475571 | chr1 | 35647557 | 35647657 | -1.45814 | eRNAcluster2 |
| chrX:47055763 | chrX | 47055763 | 47055863 | -1.43123 | eRNAcluster2 |
| chr1:356475271 | chr1 | 35647527 | 35647627 | -1.42249 | eRNAcluster2 |
| chr17:79222465 | chr17 | 79222465 | 79222565 | -1.40326 | eRNAcluster2 |
| chr6:83270039 | chr6 | 83270039 | 83270139 | -1.55025 | eRNAcluster2 |
| chr1:2345440111 | chr1 | 2.35E+08 | 2.35E+08 | -1.4001 | eRNAcluster2 |
| chr13:40932340 | chr13 | 40932340 | 40932440 | -1.50586 | eRNAcluster2 |
| chr1:356518841 | chr1 | 35651884 | 35651984 | -1.40989 | eRNAcluster2 |
| chr1:356518541 | chr1 | 35651854 | 35651954 | -1.42273 | eRNAcluster2 |
| chr1:356520541 | chr1 | 35652054 | 35652154 | -1.46894 | eRNAcluster2 |
| chr8:144677328 | chr8 | 1.45E+08 | 1.45E+08 | -1.40851 | eRNAcluster2 |
| chr3:58829815 | chr3 | 58829815 | 58829915 | -1.47825 | eRNAcluster2 |
| chr1:2345446411 | chr1 | 2.35E+08 | 2.35E+08 | -1.44864 | eRNAcluster2 |
| chr17:618762161 | chr17 | 61876216 | 61876316 | -1.40415 | eRNAcluster2 |
| chr1:2345438211 | chr1 | 2.35E+08 | 2.35E+08 | -1.44487 | eRNAcluster2 |
| chr1:2345440611 | chr1 | 2.35E+08 | 2.35E+08 | -1.4 | eRNAcluster2 |
| chr3:58829785 | chr3 | 58829785 | 58829885 | -1.51229 | eRNAcluster2 |
| chr8:98309736 | chr8 | 98309736 | 98309836 | -1.51643 | eRNAcluster2 |
| chr11:116442880 | chr11 | 1.16E+08 | 1.16E+08 | -1.50683 | eRNAcluster2 |
| chr1:356523641 | chr1 | 35652364 | 35652464 | -1.40984 | eRNAcluster2 |
| chr5:110638385 | chr5 | 1.11E+08 | 1.11E+08 | -1.41687 | eRNAcluster2 |
| chr4:120199403 | chr4 | 1.2E+08 | 1.2E+08 | -1.51386 | eRNAcluster2 |
| chr1:356516141 | chr1 | 35651614 | 35651714 | -1.40446 | eRNAcluster2 |
| chr1:2345451611 | chr1 | 2.35E+08 | 2.35E+08 | -1.40918 | eRNAcluster2 |
| chr8:144677048 | chr8 | 1.45E+08 | 1.45E+08 | -1.4518 | eRNAcluster2 |
| chr5:64009001 | chr5 | 64009001 | 64009101 | -1.4802 | eRNAcluster2 |
| chr8:26253664 | chr8 | 26253664 | 26253764 | -1.40838 | eRNAcluster2 |
| chr8:94418511 | chr8 | 94418511 | 94418611 | -1.44391 | eRNAcluster2 |
| chr13:31877339 | chr13 | 31877339 | 31877439 | -1.43305 | eRNAcluster2 |
| chr5:92550755 | chr5 | 92550755 | 92550855 | -1.45773 | eRNAcluster2 |
| chr5:110638575 | chr5 | 1.11E+08 | 1.11E+08 | -1.41099 | eRNAcluster2 |
| chr4:80889971 | chr4 | 80889971 | 80890071 | -1.48512 | eRNAcluster2 |
| chr8:62185538 | chr8 | 62185538 | 62185638 | -1.49918 | eRNAcluster2 |
| chr7:8393276 | chr7 | 8393276 | 8393376 | -1.52317 | eRNAcluster2 |
| chr4:120200373 | chr4 | 1.2E+08 | 1.2E+08 | -1.46307 | eRNAcluster2 |
| chr13:37729073 | chr13 | 37729073 | 37729173 | -1.48646 | eRNAcluster2 |
| chrX:91510413 | chrX | 91510413 | 91510513 | -1.49077 | eRNAcluster2 |
| chr7:30480782 | chr7 | 30480782 | 30480882 | -1.44018 | eRNAcluster2 |
| chr13:37728233 | chr13 | 37728233 | 37728333 | -1.42132 | eRNAcluster2 |
| chr4:80889811 | chr4 | 80889811 | 80889911 | -1.46893 | eRNAcluster2 |
| chr7:25644368 | chr7 | 25644368 | 25644468 | -1.44565 | eRNAcluster2 |
| chr8:87603540 | chr8 | 87603540 | 87603640 | -1.48219 | eRNAcluster2 |
| chr3:176545889 | chr3 | 1.77E+08 | 1.77E+08 | -1.46034 | eRNAcluster2 |
| chr1:121279517 | chr1 | 1.21E+08 | 1.21E+08 | -1.4087 | eRNAcluster2 |
| chrX:91509563 | chrX | 91509563 | 91509663 | -1.40846 | eRNAcluster2 |
| chr12:71513357 | chr12 | 71513357 | 71513457 | -1.46139 | eRNAcluster2 |
| chr4:120200403 | chr4 | 1.2E+08 | 1.2E+08 | -1.42198 | eRNAcluster2 |
| chr1:121278167 | chr1 | 1.21E+08 | 1.21E+08 | -1.4585 | eRNAcluster2 |
| chr12:96710792 | chr12 | 96710792 | 96710892 | -1.43064 | eRNAcluster2 |
| chr8:94419541 | chr8 | 94419541 | 94419641 | -1.42743 | eRNAcluster2 |
| chr5:92598655 | chr5 | 92598655 | 92598755 | -1.4424 | eRNAcluster2 |
| chr13:31877519 | chr13 | 31877519 | 31877619 | -1.41406 | eRNAcluster2 |
| chr4:80859477 | chr4 | 80859477 | 80859577 | -1.40219 | eRNAcluster2 |
| chr14:61526963 | chr14 | 61526963 | 61527063 | -1.43469 | eRNAcluster2 |
| chr12:103829666 | chr12 | 1.04E+08 | 1.04E+08 | -1.40474 | eRNAcluster2 |
| chr13:37729473 | chr13 | 37729473 | 37729573 | -1.41334 | eRNAcluster2 |
| chr14:61525963 | chr14 | 61525963 | 61526063 | -1.41313 | eRNAcluster2 |
| chr14:61526993 | chr14 | 61526993 | 61527093 | -1.41985 | eRNAcluster2 |
| chr4:80859637 | chr4 | 80859637 | 80859737 | -1.4256 | eRNAcluster2 |
| chr8:94419111 | chr8 | 94419111 | 94419211 | -1.40754 | eRNAcluster2 |
| chr5:110638885 | chr5 | 1.11E+08 | 1.11E+08 | -1.48198 | eRNAcluster2 |
| chr13:40931500 | chr13 | 40931500 | 40931600 | -1.41531 | eRNAcluster2 |
| chr1:121278137 | chr1 | 1.21E+08 | 1.21E+08 | -1.44622 | eRNAcluster2 |
| chr10:100546836 | chr10 | 1.01E+08 | 1.01E+08 | -1.41344 | eRNAcluster2 |
| chrX:28225823 | chrX | 28225823 | 28225923 | -1.43717 | eRNAcluster2 |
| chr8:98308576 | chr8 | 98308576 | 98308676 | -1.42212 | eRNAcluster2 |
| chr13:44549445 | chr13 | 44549445 | 44549545 | -1.48073 | eRNAcluster2 |
| chr3:114382266 | chr3 | 1.14E+08 | 1.14E+08 | -1.41657 | eRNAcluster2 |
| chr8:62185578 | chr8 | 62185578 | 62185678 | -1.40101 | eRNAcluster2 |
| chr1:77470145 | chr1 | 77470145 | 77470245 | -1.42693 | eRNAcluster2 |
| chr7:8393116 | chr7 | 8393116 | 8393216 | -1.41227 | eRNAcluster2 |
| chr5:145990960 | chr5 | 1.46E+08 | 1.46E+08 | -1.40641 | eRNAcluster2 |
| chr3:136686674 | chr3 | 1.37E+08 | 1.37E+08 | -1.4096 | eRNAcluster2 |
| chr8:26253874 | chr8 | 26253874 | 26253974 | -1.40342 | eRNAcluster2 |
| chr4:107496497 | chr4 | 1.07E+08 | 1.07E+08 | -1.41134 | eRNAcluster2 |
| chr18:66457038 | chr18 | 66457038 | 66457138 | -1.41794 | eRNAcluster2 |
| chr4:80888821 | chr4 | 80888821 | 80888921 | -1.40823 | eRNAcluster2 |
| chr5:92549885 | chr5 | 92549885 | 92549985 | -1.41425 | eRNAcluster2 |
| chr8:98308786 | chr8 | 98308786 | 98308886 | -1.47509 | eRNAcluster2 |
| chr5:145989970 | chr5 | 1.46E+08 | 1.46E+08 | -1.41904 | eRNAcluster2 |
| chr3:176545729 | chr3 | 1.77E+08 | 1.77E+08 | -1.42399 | eRNAcluster2 |
| chr7:30479762 | chr7 | 30479762 | 30479862 | -1.41554 | eRNAcluster2 |
| chr16:82173746 | chr16 | 82173746 | 82173846 | -1.41739 | eRNAcluster2 |
| chr9:9932287 | chr9 | 9932287 | 9932387 | -1.40124 | eRNAcluster2 |
| chr5:110638855 | chr5 | 1.11E+08 | 1.11E+08 | -1.4191 | eRNAcluster2 |
| chr3:58829555 | chr3 | 58829555 | 58829655 | -1.43802 | eRNAcluster2 |
| chr13:44548695 | chr13 | 44548695 | 44548795 | -1.41297 | eRNAcluster2 |
| chr1:121278107 | chr1 | 1.21E+08 | 1.21E+08 | -1.40021 | eRNAcluster2 |
| chr3:58827935 | chr3 | 58827935 | 58828035 | 1.837926 | eRNAcluster3 |
| chr5:92549735 | chr5 | 92549735 | 92549835 | 1.729332 | eRNAcluster3 |
| chr2:158423304 | chr2 | 1.58E+08 | 1.58E+08 | 1.718178 | eRNAcluster3 |
| chr3:588298151 | chr3 | 58829815 | 58829915 | 1.71245 | eRNAcluster3 |
| chr3:18372074 | chr3 | 18372074 | 18372174 | 1.720365 | eRNAcluster3 |
| chr16:82173596 | chr16 | 82173596 | 82173696 | 1.746778 | eRNAcluster3 |
| chr2:109925207 | chr2 | 1.1E+08 | 1.1E+08 | 1.74859 | eRNAcluster3 |
| chr13:31876509 | chr13 | 31876509 | 31876609 | 1.80447 | eRNAcluster3 |
| chr7:8343230 | chr7 | 8343230 | 8343330 | 1.714707 | eRNAcluster3 |
| chr8:262538741 | chr8 | 26253874 | 26253974 | 1.73374 | eRNAcluster3 |
| chr4:107495557 | chr4 | 1.07E+08 | 1.07E+08 | 1.763054 | eRNAcluster3 |
| chr6:83269689 | chr6 | 83269689 | 83269789 | 1.754573 | eRNAcluster3 |
| chr6:113240891 | chr6 | 1.13E+08 | 1.13E+08 | 1.731537 | eRNAcluster3 |
| chr3:58829755 | chr3 | 58829755 | 58829855 | 1.735457 | eRNAcluster3 |
| chr7:8340920 | chr7 | 8340920 | 8341020 | 1.751734 | eRNAcluster3 |
| chr3:588295551 | chr3 | 58829555 | 58829655 | 1.824207 | eRNAcluster3 |
| chr9:9931947 | chr9 | 9931947 | 9932047 | 1.731891 | eRNAcluster3 |
| chr1:121279857 | chr1 | 1.21E+08 | 1.21E+08 | 1.720986 | eRNAcluster3 |
| chr6:83269789 | chr6 | 83269789 | 83269889 | 1.793013 | eRNAcluster3 |
| chr4:169363903 | chr4 | 1.69E+08 | 1.69E+08 | 1.709836 | eRNAcluster3 |
| chr7:83932761 | chr7 | 8393276 | 8393376 | 1.779255 | eRNAcluster3 |
| chr8:983085761 | chr8 | 98308576 | 98308676 | 1.734177 | eRNAcluster3 |
| chr8:621855781 | chr8 | 62185578 | 62185678 | 1.710475 | eRNAcluster3 |
| chrX:28225613 | chrX | 28225613 | 28225713 | 1.719029 | eRNAcluster3 |
| chr7:83931161 | chr7 | 8393116 | 8393216 | 1.72675 | eRNAcluster3 |
| chr1:121279657 | chr1 | 1.21E+08 | 1.21E+08 | 1.744664 | eRNAcluster3 |
| chr8:983097361 | chr8 | 98309736 | 98309836 | 1.734417 | eRNAcluster3 |
| chrX:28227053 | chrX | 28227053 | 28227153 | 1.713597 | eRNAcluster3 |
| chr3:588297851 | chr3 | 58829785 | 58829885 | 1.722678 | eRNAcluster3 |
| chr5:90224756 | chr5 | 90224756 | 90224856 | 1.701949 | eRNAcluster3 |
| chr3:62836296 | chr3 | 62836296 | 62836396 | 1.789107 | eRNAcluster3 |
| chr15:53834500 | chr15 | 53834500 | 53834600 | 1.757367 | eRNAcluster3 |
| chr4:123577176 | chr4 | 1.24E+08 | 1.24E+08 | 1.746994 | eRNAcluster3 |
| chr7:114669616 | chr7 | 1.15E+08 | 1.15E+08 | 1.727593 | eRNAcluster3 |
| chr7:30457278 | chr7 | 30457278 | 30457378 | 1.702449 | eRNAcluster3 |
| chr16:821737461 | chr16 | 82173746 | 82173846 | 1.747519 | eRNAcluster3 |
| chr12:103829836 | chr12 | 1.04E+08 | 1.04E+08 | 1.73461 | eRNAcluster3 |
| chr3:1143822661 | chr3 | 1.14E+08 | 1.14E+08 | 1.710104 | eRNAcluster3 |
| chr11:116445690 | chr11 | 1.16E+08 | 1.16E+08 | 1.751798 | eRNAcluster3 |
| chr11:1164428801 | chr11 | 1.16E+08 | 1.16E+08 | 1.702832 | eRNAcluster3 |
| chr4:808888211 | chr4 | 80888821 | 80888921 | 1.707724 | eRNAcluster3 |
| chr4:107498197 | chr4 | 1.07E+08 | 1.07E+08 | 1.741287 | eRNAcluster3 |
| chr5:41237915 | chr5 | 41237915 | 41238015 | 1.703155 | eRNAcluster3 |
| chr12:85709392 | chr12 | 85709392 | 85709492 | 1.777437 | eRNAcluster3 |
| chr7:114669516 | chr7 | 1.15E+08 | 1.15E+08 | 1.73908 | eRNAcluster3 |
| chr5:110639055 | chr5 | 1.11E+08 | 1.11E+08 | 1.759375 | eRNAcluster3 |
| chr13:31879139 | chr13 | 31879139 | 31879239 | 1.734496 | eRNAcluster3 |
| chr2:233222669 | chr2 | 2.33E+08 | 2.33E+08 | 1.77487 | eRNAcluster3 |
| chr1:58597436 | chr1 | 58597436 | 58597536 | 1.732988 | eRNAcluster3 |
| chr4:169366033 | chr4 | 1.69E+08 | 1.69E+08 | 1.733411 | eRNAcluster3 |
| chr6:8568117 | chr6 | 8568117 | 8568217 | 1.733602 | eRNAcluster3 |
| chr4:80889911 | chr4 | 80889911 | 80890011 | 1.731778 | eRNAcluster3 |
| chr8:26255844 | chr8 | 26255844 | 26255944 | 1.760838 | eRNAcluster3 |
| chr11:116442640 | chr11 | 1.16E+08 | 1.16E+08 | 1.720906 | eRNAcluster3 |
| chr5:57586599 | chr5 | 57586599 | 57586699 | 1.772842 | eRNAcluster3 |
| chr13:31878419 | chr13 | 31878419 | 31878519 | 1.758188 | eRNAcluster3 |
| chr13:445486951 | chr13 | 44548695 | 44548795 | 1.744335 | eRNAcluster3 |
| chr3:1765457291 | chr3 | 1.77E+08 | 1.77E+08 | 1.722387 | eRNAcluster3 |
| chr20:18934687 | chr20 | 18934687 | 18934787 | 1.777276 | eRNAcluster3 |
| chrX:28226553 | chrX | 28226553 | 28226653 | 1.722207 | eRNAcluster3 |
| chr5:64007611 | chr5 | 64007611 | 64007711 | 1.861696 | eRNAcluster3 |
| chr13:31879749 | chr13 | 31879749 | 31879849 | 1.867092 | eRNAcluster3 |
| chrX:28227603 | chrX | 28227603 | 28227703 | 1.753523 | eRNAcluster3 |
| chr11:7884250 | chr11 | 7884250 | 7884350 | 1.712957 | eRNAcluster3 |
| chr8:98310556 | chr8 | 98310556 | 98310656 | 1.753668 | eRNAcluster3 |
| chr5:92598415 | chr5 | 92598415 | 92598515 | 1.738742 | eRNAcluster3 |
| chr12:85711112 | chr12 | 85711112 | 85711212 | 1.766967 | eRNAcluster3 |
| chr5:1106388551 | chr5 | 1.11E+08 | 1.11E+08 | 1.7219 | eRNAcluster3 |
| chr2:158424854 | chr2 | 1.58E+08 | 1.58E+08 | 1.812606 | eRNAcluster3 |
| chr11:116444390 | chr11 | 1.16E+08 | 1.16E+08 | 1.762862 | eRNAcluster3 |
| chr1:1212781071 | chr1 | 1.21E+08 | 1.21E+08 | 1.79339 | eRNAcluster3 |
| chr5:92550525 | chr5 | 92550525 | 92550625 | 1.726579 | eRNAcluster3 |
| chr1:121276667 | chr1 | 1.21E+08 | 1.21E+08 | 1.76808 | eRNAcluster3 |
| chr1:121276997 | chr1 | 1.21E+08 | 1.21E+08 | 1.703022 | eRNAcluster3 |
| chr7:25644608 | chr7 | 25644608 | 25644708 | 1.721759 | eRNAcluster3 |
| chr3:136686614 | chr3 | 1.37E+08 | 1.37E+08 | 1.775093 | eRNAcluster3 |
| chr4:120198573 | chr4 | 1.2E+08 | 1.2E+08 | 1.729626 | eRNAcluster3 |
| chr4:80890011 | chr4 | 80890011 | 80890111 | 1.700078 | eRNAcluster3 |
| chr3:62835696 | chr3 | 62835696 | 62835796 | 1.838565 | eRNAcluster3 |
| chr11:116444260 | chr11 | 1.16E+08 | 1.16E+08 | 1.87132 | eRNAcluster3 |
| chr6:75195691 | chr6 | 75195691 | 75195791 | 1.732334 | eRNAcluster3 |
| chr14:61524593 | chr14 | 61524593 | 61524693 | 1.834214 | eRNAcluster3 |
| chr1:58595436 | chr1 | 58595436 | 58595536 | 1.700677 | eRNAcluster3 |
| chr5:145991020 | chr5 | 1.46E+08 | 1.46E+08 | 1.757153 | eRNAcluster3 |
| chr18:47674492 | chr18 | 47674492 | 47674592 | 1.888728 | eRNAcluster3 |
| chr2:118382496 | chr2 | 1.18E+08 | 1.18E+08 | 1.722604 | eRNAcluster3 |
| chr6:113238891 | chr6 | 1.13E+08 | 1.13E+08 | 1.71149 | eRNAcluster3 |
| chr20:18933367 | chr20 | 18933367 | 18933467 | 1.831505 | eRNAcluster3 |
| chr2:74729657 | chr2 | 1.1E+08 | 1.1E+08 | 1.738911 | eRNAcluster3 |
| chr7:25643798 | chr7 | 25643798 | 25643898 | 1.706052 | eRNAcluster3 |
| chr5:110639905 | chr5 | 1.11E+08 | 1.11E+08 | 1.731304 | eRNAcluster3 |
| chr10:100546776 | chr10 | 1.01E+08 | 1.01E+08 | 1.755487 | eRNAcluster3 |
| chr4:107495387 | chr4 | 1.07E+08 | 1.07E+08 | 1.708585 | eRNAcluster3 |
| chr4:80862157 | chr4 | 80862157 | 80862257 | 1.796418 | eRNAcluster3 |
| chr2:158424754 | chr2 | 1.58E+08 | 1.58E+08 | 1.703853 | eRNAcluster3 |
| chr4:107496277 | chr4 | 1.07E+08 | 1.07E+08 | 1.767475 | eRNAcluster3 |
| chr6:55513437 | chr6 | 55513437 | 55513537 | 1.780944 | eRNAcluster3 |
| chr4:80891341 | chr4 | 80891341 | 80891441 | 1.804378 | eRNAcluster3 |
| chr9:135402163 | chr9 | 1.35E+08 | 1.35E+08 | 1.810988 | eRNAcluster3 |
| chr3:106696920 | chr3 | 1.07E+08 | 1.07E+08 | 1.718982 | eRNAcluster3 |
| chr11:92908937 | chr11 | 92908937 | 92909037 | 1.76016 | eRNAcluster3 |
| chr1:174815108 | chr1 | 1.75E+08 | 1.75E+08 | 1.81929 | eRNAcluster3 |
| chr14:61524723 | chr14 | 61524723 | 61524823 | 1.718577 | eRNAcluster3 |
| chr8:983087861 | chr8 | 98308786 | 98308886 | 1.729974 | eRNAcluster3 |
| chr13:445494451 | chr13 | 44549445 | 44549545 | 1.715188 | eRNAcluster3 |
| chr5:110641215 | chr5 | 1.11E+08 | 1.11E+08 | 1.809299 | eRNAcluster3 |
| chr2:158426144 | chr2 | 1.58E+08 | 1.58E+08 | 1.767404 | eRNAcluster3 |
| chr8:98308996 | chr8 | 98308996 | 98309096 | 1.722187 | eRNAcluster3 |
| chr7:25645368 | chr7 | 25645368 | 25645468 | 1.710843 | eRNAcluster3 |
| chr4:107494957 | chr4 | 1.07E+08 | 1.07E+08 | 1.836955 | eRNAcluster3 |
| chr13:44547065 | chr13 | 44547065 | 44547165 | 1.865264 | eRNAcluster3 |
| chr4:107496247 | chr4 | 1.07E+08 | 1.07E+08 | 1.736602 | eRNAcluster3 |
| chr3:106697020 | chr3 | 1.07E+08 | 1.07E+08 | 1.727382 | eRNAcluster3 |
| chr3:62835576 | chr3 | 62835576 | 62835676 | 1.727033 | eRNAcluster3 |
| chr7:30482142 | chr7 | 30482142 | 30482242 | 1.75262 | eRNAcluster3 |
| chr5:145992330 | chr5 | 1.46E+08 | 1.46E+08 | 1.754015 | eRNAcluster3 |
| chrX:28228163 | chrX | 28228163 | 28228263 | 1.794225 | eRNAcluster3 |
| chr2:158425584 | chr2 | 1.58E+08 | 1.58E+08 | 1.707273 | eRNAcluster3 |
| chr3:136685294 | chr3 | 1.37E+08 | 1.37E+08 | 1.80565 | eRNAcluster3 |
| chr13:44547175 | chr13 | 44547175 | 44547275 | 1.732106 | eRNAcluster3 |
| chr1:121276787 | chr1 | 1.21E+08 | 1.21E+08 | 1.801609 | eRNAcluster3 |
| chr3:1931169521 | chr3 | 1.93E+08 | 1.93E+08 | -1.82412 | eRNAcluster3 |
| chr1:231999176 | chr1 | 2.32E+08 | 2.32E+08 | -1.79679 | eRNAcluster3 |
| chr12:116448960 | chr12 | 1.16E+08 | 1.16E+08 | -1.79759 | eRNAcluster3 |

| **Table S4.** SE-regulated genes in each cluster. | | |
| --- | --- | --- |
| **cluster 1** | **cluster 2** | **cluster 3** |
| ECHDC2 | CHST7 | CCDC148 |
| SCP2 | SLC9A7 | PKP4 |
| PODN | RP2 | DAPL1 |
| SLC1A7 | JADE3 | SATB1 |
| CPT2 | RGN | SEPTIN10 |
| CZIB | NDUFB11 | SOWAHC |
| MAGOH | RBM10 | RGPD5 |
| LRP8 | UBA1 | LIMS3 |
| DMRTB1 | CDK16 | TNN |
| GLIS1 | USP11 | MALL |
| NDC1 | ZNF157 | NPHP1 |
| YIPF1 | ZNF41 | MTLN |
| SFPQ | USP36 | PFKM |
| ZMYM4 | TIMP2 | SH3RF1 |
| KIAA0319L | CEP295NL | NEK1 |
| NCDN | LGALS3BP | CLCN3 |
| TFAP2E | CANT1 | HPF1 |
| PSMB2 | C1QTNF1 | CETN3 |
| C1orf216 | ENGASE | MBLAC2 |
| CLSPN | RBFOX3 | NUP98 |
| AGO4 | UBE3D | HUP153 |
| AGO1 | DOP1A | GCK |
| AGO3 | PGM3 | HIF1A |
| TEKT2 | RWDD2A | PRKAG2 |
| ADPRS | ME1 | SYNPR |
| COL8A2 | PRSS35 | WDR72 |
| TRAPPC3 | SNAP91 | P2RX7 |
| KCNE1 | FOXO1 | SPRY1 |
| RCAN1 | MRPS31 | MDFIC |
| CLIC6 | SLC25A15 | PRKAA1 |
| RUNX1 | ELF1 | RPL37 |
| COA6 | WBP4 | CARD6 |
| TARBP1 | KBTBD6 | C7 |
| IRF2BP2 | KBTBD7 | INSR |
| TBX2 | MTRF1 | MET |
| TBX4 | NAA16 | PLCXD3 |
| NACA2 | BOP1 | OXCT1 |
| BRIP1 | SCX | ALX1 |
| INTS2 | HSF1 | RASSF9 |
| MED13 | DGAT1 | KLF4A |
| EFCAB3 | SCRT1 | MGAT4C |
| DESI2 | SLC52A2 | HK2 |
| AL451007.3 | TMEM249 | SNORC |
| COX20 | AC233992.2 | NGEF |
| HNRNPU | FBXL6 | NEU2 |
| EFCAB2 | ADCK5 | INPP5D |
| KIF26B | CPSF1 | INS |
| SSTR2 | SLC39A4 | SAG |
| COG1 | VPS28 | DGKD |
| FAM104A | TONSL | USP40 |
| C17orf80 | CYHR1 | AP2B1 |
| CPSF4L | KIFC2 | ASAP1 |
| CDC42EP4 | FOXH1 | UGT1A9 |
| SDK2 | PPP1R16A | UGT1A7 |
| TEX22 | GPT | UGT1A6 |
| MTA1 | MFSD3 | CYFIP1 |
| CRIP2 | RECQL4 | UGT1A4 |
| CRIP1 | LRRC14 | OMA1 |
| AL928654.3 | LRRC24 | TACSTD2 |
| TEDC1 | C8orf82 | MYSM1 |
| TMEM121 | ARHGAP39 | CDC27 |
| EMP2 | ZNF251 | EEF1E1 |
| TEKT5 | ZNF34 | SLC2A1 |
| NUBP1 | RPL8 | GPBP1 |
| TVP23A | ZNF517 | ACTBL2 |
| CIITA | ZNF7 | POLR3F |
| DEXI | COMMD5 | RBBP9 |
| CLEC16A | ZNF250 | SEC23B |
| RMI2 | ZNF16 | SMIM26 |
| SOCS1 | C8orf33 | OGT |
| TNP2 | PXK | DTD1 |
| PRM3 | PDHB | SCP2D1 |
| PRM2 | KCTD6 | SLC24A3 |
| PRM1 | ACOX2 | OLFML1 |
| AC099489.1 | FAM107A | PPFIBP2 |
| F7 | FAM3D | CYB5R2 |
| F10 | CFAP20DC | OVCH2 |
| PROZ | MATN2 | OR5P2 |
| PCID2 | RPL30 | OR5P3 |
| CUL4A | ERICH5 | OR10A6 |
| LAMP1 | RIDA | OR10A3 |
| GRTP1 | POP1 | NLRP10 |
| ADPRHL1 | NIPAL2 | EIF3F |
| DCUN1D2 | STK3 | TUB |
| TMCO3 | KCNS2 | RIC3 |
| TFDP1 | BUD13 | LMO1 |
| ATP4B | ZPR1 | COL12A1 |
| GRK1 | APOA5 | COX7A2 |
| TMEM255B | APOA4 | TMEM30A |
| GAS6 | APOC3 | FILIP1 |
| C13orf46 | APOA1 | SENP6 |
| RASA3 | SIK3 | SKOR2 |
| TANK | TMEM232 | SMAD2 |
| PSMD14 | SLC25A46 | ZBTB7C |
| TBR1 | TSLP | CCDC93 |
| SLC4A10 | WDR36 | INSIG2 |
|  | MAD2L1 | ENO1 |
|  | PRDM5 | HCRTR2 |
|  | HTR1A | GFRAL |
|  | RNF180 | HMGCLL1 |
|  | RGS7BP | BMP5 |
|  | EBF2 | FCN1 |
|  | PPP2R2A | OLFM1 |
|  | BNIP3L | PPP1R26 |
|  | PNMA2 | HK2P1 |
|  | DPYSL2 | MRPS2 |
|  | ADRA1A | LCN1 |
|  | CDH17 | OBP2A |
|  | GEM | PAEP |
|  | RAD54B | PRKAA1 |
|  | FSBP | GLT6D1 |
|  | VIRMA | LCN9 |
|  | ESRP1 | SOHLH1 |
|  | DPY19L4 | KCNT1 |
|  | INTS8 | CAMSAP1 |
|  | CCNE2 | MTNR1B |
|  | NDUFAF6 | SLC36A4 |
|  | RXFP2 | ALDOB |
|  | FRY | KCNJ13 |
|  | ZAR1L | CACYBP |
|  | BRCA2 | HK1 |
|  | BMP3 | AL354761.1 |
|  | PRKG2 |  |
|  | NKAIN3 |  |
|  | GLCCI1 |  |
|  | ICA1 |  |
|  | NXPH1 |  |
|  | POSTN |  |
|  | TRPC4 |  |
|  | PABPC5 |  |
|  | PCDH11X |  |
|  | FKBP14 |  |
|  | PLEKHA8 |  |
|  | MTURN |  |
|  | ZNRF2 |  |
|  | AC006978.2 |  |
|  | NOD1 |  |
|  | GGCT |  |
|  | AC005154.5 |  |
|  | GARS1 |  |
|  | CRHR2 |  |
|  | INMT |  |
|  | INMT-MINDY4 |  |
|  | MINDY4 |  |
|  | AC004691.2 |  |
|  | AQP1 |  |
|  | GHRHR |  |
|  | NPVF |  |
|  | DCAF4L2 |  |
|  | MMP16 |  |
|  | TBL1XR1 |  |
|  | PPIAL4A |  |
|  | FCGR1B |  |
|  | FAM72B |  |
|  | SRGAP2C |  |
|  | TSPAN8 |  |
|  | LGR5 |  |
|  | ZFC3H1 |  |
|  | THAP2 |  |
|  | AC073612.1 |  |
|  | TMEM19 |  |
|  | RAB21 |  |
|  | TBC1D15 |  |
|  | TPH2 |  |
|  | CDK17 |  |
|  | CFAP54 |  |
|  | NEDD1 |  |
|  | PRKCH |  |
|  | TMEM30B |  |
|  | AL355916.3 |  |
|  | HIF1A |  |
|  | SNAPC1 |  |
|  | SYT16 |  |
|  | STAB2 |  |
|  | NT5DC3 |  |
|  | HSP90B1 |  |
|  | C12orf73 |  |
|  | TDG |  |
|  | GLT8D2 |  |
|  | HCFC2 |  |
|  | NFYB |  |
|  | TXNRD1 |  |
|  | EID3 |  |
|  | ERLIN1 |  |
|  | CHUK |  |
|  | CWF19L1 |  |
|  | BLOC1S2 |  |
|  | PKD2L1 |  |
|  | SCD |  |
|  | WNT8B |  |
|  | SEC31B |  |
|  | AL133352.1 |  |
|  | NDUFB8 |  |
|  | HIF1AN |  |
|  | PAX2 |  |
|  | SLF2 |  |
|  | MRPL43 |  |
|  | SEMA4G |  |
|  | TWNK |  |
|  | LZTS2 |  |
|  | PDZD7 |  |
|  | SFXN3 |  |
|  | MAGEB10 |  |
|  | DCAF8L1 |  |
|  | IL1RAPL1 |  |
|  | SMIM2 |  |
|  | SERP2 |  |
|  | TSC22D1 |  |
|  | NUFIP1 |  |
|  | GPALPP1 |  |
|  | ZDHHC23 |  |
|  | CCDC191 |  |
|  | QTRT2 |  |
|  | DRD3 |  |
|  | ZNF80 |  |
|  | TIGIT |  |
|  | ZBTB20 |  |
|  | PIGK |  |
|  | AK5 |  |
|  | ZZZ3 |  |
|  | USP33 |  |
|  | MIGA1 |  |
|  | NEXN |  |
|  | FUBP1 |  |
|  | GRXCR2 |  |
|  | SH3RF2 |  |
|  | PLAC8L1 |  |
|  | LARS1 |  |
|  | AC091959.1 |  |

| **Table S5** The overlap genes between SPGs and DEGs in each cluster | | | | |
| --- | --- | --- | --- | --- |
| symbol | Median | | | cluster |
|  | cluster.1 | cluster.2 | cluster.3 |  |
| ECHDC2 | 11.87171 | 10.92585 | 11.13296 | 1 |
| PODN | 11.07065 | 10.61333 | 10.43835 | 1 |
| SLC1A7 | 8.196091 | 7.143626 | 6.460488 | 1 |
| CZIB | 10.62025 | 10.53255 | 10.29501 | 1 |
| LRP8 | 9.621591 | 9.329594 | 9.514022 | 1 |
| SFPQ | 13.64022 | 13.41822 | 13.604 | 1 |
| KIAA0319L | 12.77661 | 12.51487 | 12.45192 | 1 |
| NCDN | 10.89442 | 10.70918 | 10.64067 | 1 |
| TFAP2E | 7.131006 | 6.422218 | 6.650338 | 1 |
| C1orf216 | 9.843684 | 9.607433 | 9.553136 | 1 |
| CLSPN | 8.785384 | 7.989377 | 8.72907 | 1 |
| AGO4 | 10.90062 | 10.46258 | 10.57758 | 1 |
| AGO1 | 11.02577 | 10.93276 | 11.06276 | 1 |
| AGO3 | 10.36545 | 9.923811 | 10.10561 | 1 |
| TEKT2 | 6.84996 | 6.46057 | 6.189893 | 1 |
| COL8A2 | 10.1539 | 10.06509 | 9.21905 | 1 |
| TRAPPC3 | 11.59282 | 11.49353 | 11.23406 | 1 |
| KCNE1 | 5.804753 | 5.438236 | 5.212104 | 1 |
| CLIC6 | 12.83635 | 12.10595 | 12.03386 | 1 |
| RUNX1 | 12.79533 | 12.69299 | 12.34986 | 1 |
| TARBP1 | 11.44687 | 10.67148 | 11.08109 | 1 |
| IRF2BP2 | 13.01371 | 12.91122 | 12.99396 | 1 |
| TBX2 | 10.23987 | 9.80094 | 9.594648 | 1 |
| TBX4 | 8.718344 | 8.500173 | 8.125304 | 1 |
| NACA2 | 4.90446 | 4.661037 | 4.571751 | 1 |
| MED13 | 12.99075 | 11.9244 | 12.09751 | 1 |
| EFCAB2 | 9.436709 | 9.121397 | 9.395064 | 1 |
| SDK2 | 7.976081 | 7.624755 | 7.347093 | 1 |
| TEX22 | 5.620842 | 5.163698 | 5.322412 | 1 |
| EMP2 | 13.49021 | 13.37307 | 12.82132 | 1 |
| TEKT5 | 5.331572 | 5.121666 | 5.092883 | 1 |
| TVP23A | 7.048275 | 6.582014 | 6.326552 | 1 |
| CIITA | 11.6109 | 11.01583 | 10.11415 | 1 |
| DEXI | 8.155264 | 8.13857 | 8.058739 | 1 |
| CLEC16A | 11.11041 | 10.91038 | 10.95029 | 1 |
| DCUN1D2 | 9.093913 | 8.755268 | 9.00635 | 1 |
| ATP4B | 4.875513 | 4.438927 | 4.604638 | 1 |
| GRK1 | 4.882475 | 4.481593 | 4.623992 | 1 |
| GAS6 | 12.15518 | 12.00632 | 11.43984 | 1 |
| ARMH2 | 4.029923 | 3.956005 | 3.600891 | 1 |
| ATP13A4 | 11.04392 | 9.946103 | 8.499751 | 1 |
| RBM10 | 11.47164 | 11.34232 | 11.70932 | 2 |
| CDK16 | 12.02846 | 11.14435 | 12.60416 | 2 |
| USP11 | 11.68568 | 11.65678 | 11.90503 | 2 |
| ZNF157 | 4.586618 | 4.372309 | 4.730373 | 2 |
| USP36 | 11.54106 | 11.33006 | 11.56948 | 2 |
| CEP295NL | 4.631417 | 4.564914 | 4.673699 | 2 |
| ENGASE | 11.09883 | 9.893573 | 10.37346 | 2 |
| RBFOX3 | 4.822022 | 4.435452 | 4.604683 | 2 |
| DOP1A | 9.860282 | 9.406677 | 9.537186 | 2 |
| SNAP91 | 4.501914 | 4.401085 | 4.563569 | 2 |
| MTRF1 | 8.623622 | 8.337872 | 8.520728 | 2 |
| NAA16 | 9.374469 | 8.959942 | 9.184421 | 2 |
| SCX | 6.630328 | 6.237958 | 6.447625 | 2 |
| SCRT1 | 4.364469 | 4.17189 | 4.324949 | 2 |
| TMEM249 | 4.681989 | 4.441664 | 4.547565 | 2 |
| FBXL6 | 10.18857 | 9.949312 | 10.06654 | 2 |
| ADCK5 | 9.365786 | 8.985272 | 9.435347 | 2 |
| CPSF1 | 12.3347 | 11.95371 | 12.5198 | 2 |
| TONSL | 9.817945 | 9.621596 | 10.39716 | 2 |
| CYHR1 | 11.50702 | 11.198 | 11.51327 | 2 |
| KIFC2 | 9.979519 | 9.066144 | 9.819663 | 2 |
| FOXH1 | 4.712389 | 4.449223 | 4.795013 | 2 |
| PPP1R16A | 10.38604 | 10.29083 | 10.46102 | 2 |
| GPT | 6.958406 | 6.308041 | 7.06819 | 2 |
| RECQL4 | 9.758225 | 9.55764 | 10.47643 | 2 |
| LRRC14 | 10.67056 | 10.29213 | 10.65681 | 2 |
| ARHGAP39 | 9.67772 | 9.241854 | 10.09236 | 2 |
| ZNF251 | 9.898551 | 9.445127 | 9.749258 | 2 |
| ZNF34 | 8.18612 | 8.115621 | 8.227386 | 2 |
| ZNF517 | 9.343234 | 9.027389 | 9.387495 | 2 |
| ZNF7 | 10.12425 | 9.954586 | 10.21864 | 2 |
| ZNF250 | 9.103411 | 8.924596 | 9.1462 | 2 |
| ZNF16 | 8.796239 | 8.883512 | 9.037542 | 2 |
| CFAP20DC | 7.29083 | 7.174563 | 7.635009 | 2 |
| MATN2 | 9.448063 | 9.245462 | 9.615464 | 2 |
| BUD13 | 9.808463 | 9.922453 | 10.00048 | 2 |
| APOA5 | 3.952216 | 3.600891 | 4.051021 | 2 |
| SIK3 | 10.94664 | 10.82533 | 10.90276 | 2 |
| TSLP | 5.338562 | 5.26969 | 4.800516 | 2 |
| WDR36 | 10.56151 | 10.68918 | 10.54542 | 2 |
| EBF2 | 5.91467 | 5.792989 | 5.823292 | 2 |
| CCNE2 | 7.606905 | 7.57073 | 8.065079 | 2 |
| ZAR1L | 4.158179 | 4.088376 | 4.257082 | 2 |
| ICA1 | 11.04345 | 10.92095 | 11.33664 | 2 |
| NXPH1 | 4.075209 | 4.058939 | 4.584422 | 2 |
| PLEKHA8 | 9.945714 | 9.698999 | 9.94849 | 2 |
| CRHR2 | 5.178829 | 4.779056 | 5.074417 | 2 |
| ZFC3H1 | 10.91403 | 10.36996 | 10.8284 | 2 |
| WNT8B | 4.846189 | 4.494328 | 4.809941 | 2 |
| SEC31B | 9.016109 | 7.574881 | 7.937123 | 2 |
| HIF1AN | 11.28139 | 11.21652 | 11.36726 | 2 |
| SEMA4G | 8.856537 | 8.637814 | 9.184278 | 2 |
| LZTS2 | 11.74265 | 11.65867 | 11.7527 | 2 |
| ZDHHC23 | 9.085654 | 8.720897 | 9.379836 | 2 |
| CCDC191 | 9.074203 | 8.354493 | 8.413502 | 2 |
| QTRT2 | 10.04843 | 10.01513 | 10.1723 | 2 |
| FUBP1 | 12.06495 | 12.01146 | 12.33147 | 2 |
| PLAC8L1 | 4.750043 | 4.517317 | 4.649457 | 2 |
| LARS1 | 12.0474 | 11.47699 | 12.11249 | 2 |
| POU4F3 | 4.147969 | 4.144916 | 4.178284 | 2 |
| TCERG1 | 11.21993 | 10.93156 | 11.34254 | 2 |
| PCCB | 10.5103 | 9.888197 | 11.00975 | 2 |
| STAG1 | 10.01261 | 9.170939 | 10.29574 | 2 |
| SLC35G2 | 7.677582 | 7.093457 | 7.870611 | 2 |
| NCK1 | 10.17747 | 10.00954 | 10.19587 | 2 |
| IL20RB | 6.416566 | 6.275254 | 6.914315 | 2 |
| PAPSS1 | 11.27576 | 11.03358 | 11.33244 | 2 |
| SGMS2 | 11.55218 | 10.68906 | 11.01336 | 2 |
| CYP2U1 | 8.876272 | 7.936945 | 8.412836 | 2 |
| HADH | 11.04274 | 10.20184 | 10.95672 | 2 |
| CDH19 | 4.909104 | 4.410311 | 4.566216 | 2 |
| PLCG2 | 10.4645 | 9.024477 | 9.91174 | 2 |
| SDR42E1 | 9.065021 | 8.929032 | 9.20638 | 2 |
| HSD17B2 | 5.865913 | 5.505315 | 5.618124 | 2 |
| MPHOSPH6 | 9.128125 | 9.004607 | 9.427396 | 2 |
| CDH13 | 8.547671 | 8.233113 | 8.463014 | 2 |
| CCDC148 | 6.105061 | 6.138484 | 6.533869 | 3 |
| PKP4 | 11.85786 | 11.75572 | 12.02487 | 3 |
| SATB1 | 10.32595 | 9.932593 | 10.44992 | 3 |
| LIMS3 | 3.600891 | 3.600891 | 3.600891 | 3 |
| MALL | 10.20246 | 9.938094 | 10.2576 | 3 |
| NEK1 | 9.233066 | 9.269086 | 9.946698 | 3 |
| MBLAC2 | 8.369047 | 8.345903 | 8.665329 | 3 |
| POLR3G | 7.150504 | 7.487755 | 7.586461 | 3 |
| LYSMD3 | 10.42147 | 10.56393 | 10.90966 | 3 |
| ADGRV1 | 9.533638 | 8.638892 | 9.801697 | 3 |
| CADPS | 5.355445 | 5.183241 | 5.979389 | 3 |
| WDR72 | 6.445063 | 6.364739 | 9.749618 | 3 |
| UNC13C | 4.62512 | 4.53387 | 5.218931 | 3 |
| MDFIC | 11.41808 | 11.6406 | 11.75414 | 3 |
| C7 | 11.80364 | 11.25061 | 12.23836 | 3 |
| MET | 12.93337 | 13.24829 | 14.00948 | 3 |
| PLCXD3 | 6.521285 | 6.343363 | 6.771308 | 3 |
| OXCT1 | 9.98303 | 9.981086 | 10.83661 | 3 |
| NGEF | 8.273762 | 8.421347 | 8.491653 | 3 |
| INPP5D | 11.0254 | 10.67379 | 11.30393 | 3 |
| ATG16L1 | 10.746 | 10.82744 | 11.25634 | 3 |
| SAG | 4.146443 | 4.140774 | 4.467125 | 3 |
| DGKD | 11.77638 | 11.10549 | 11.92355 | 3 |
| USP40 | 11.43352 | 11.32055 | 11.93076 | 3 |
| AP2B1 | 13.48002 | 13.65945 | 13.62343 | 3 |
| ASAP1 | 10.91124 | 11.08914 | 11.37258 | 3 |
| CYFIP1 | 12.47714 | 12.71315 | 12.88641 | 3 |
| MYSM1 | 10.76352 | 10.11076 | 10.86724 | 3 |
| SLC2A1 | 11.42711 | 12.42483 | 12.87329 | 3 |
| RBBP9 | 10.374 | 10.56043 | 10.58596 | 3 |
| SEC23B | 11.89806 | 12.0366 | 12.22283 | 3 |
| SLC24A3 | 8.301607 | 8.265359 | 8.577366 | 3 |
| PPFIBP2 | 10.58803 | 10.246 | 11.20603 | 3 |
| CYB5R2 | 8.604441 | 8.572725 | 8.781832 | 3 |
| NLRP10 | 3.600891 | 3.600891 | 3.964122 | 3 |
| TUB | 8.349474 | 7.699874 | 8.772451 | 3 |
| LMO1 | 4.220161 | 4.215301 | 4.511793 | 3 |
| COX7A2 | 11.41042 | 11.9166 | 12.05611 | 3 |
| TMEM30A | 13.23895 | 13.53596 | 13.65714 | 3 |
| SENP6 | 11.5148 | 11.39867 | 11.58728 | 3 |
| SKOR2 | 4.24204 | 3.92423 | 4.642103 | 3 |
| SMAD2 | 11.7454 | 11.91457 | 11.95381 | 3 |
| ZBTB7C | 9.048292 | 9.011624 | 9.93685 | 3 |
| CCDC93 | 10.03292 | 10.60497 | 10.73567 | 3 |
| INSIG2 | 10.64296 | 10.74969 | 10.90069 | 3 |
| ENO1 | 16.13146 | 16.11221 | 16.19731 | 3 |
| OLFM1 | 7.01941 | 8.632158 | 9.340147 | 3 |
| KCNT1 | 5.206258 | 4.712491 | 6.013036 | 3 |
| ALDOB | 5.421156 | 5.223222 | 5.864167 | 3 |
| HK2 | 11.29394 | 11.67212 | 11.76938 | 3 |
| HK1 | 12.63156 | 12.83106 | 13.02862 | 3 |
| PFKM | 10.82882 | 10.75867 | 11.24082 | 3 |
| INSR | 11.58346 | 11.61119 | 11.84002 | 3 |
| INS | 4.74272 | 4.413219 | 5.08943 | 3 |
| OGT | 13.46817 | 12.48982 | 13.70849 | 3 |
| NUP98 | 12.17025 | 12.30705 | 12.42594 | 3 |
| GCK | 4.740671 | 4.730242 | 4.792401 | 3 |
| HIF1A | 12.8634 | 13.32859 | 13.602 | 3 |
| PRKAG2 | 10.30431 | 10.41005 | 10.65375 | 3 |
| KDM3A | 8.41335 | 8.31421 | 8.94824 | 3 |
| CDC27 | 7.93203 | 8.01324 | 8.42303 | 3 |

| **Table S3.** Identified TFs with potential molecular function in promoters of SE-regulated genes in each cluster. | |
| --- | --- |
| **symbol** | **ENSEMBL** |
| MSANTD3 | ENSG00000066697 |
| BATF3 | ENSG00000123685 |
| FOS | ENSG00000170345 |
| FOSL1 | ENSG00000175592 |
| JUN | ENSG00000177606 |
| PBX1 | ENSG00000185630 |
| JDP1 | ENSG00000108176 |
| SPI1 | ENSG00000066336 |
| HOXB5 | ENSG00000120075 |
| PRDM14 | ENSG00000147596 |
| HOXA2 | ENSG00000105996 |
| HOXB4 | ENSG00000182742 |
| HOXD3 | ENSG00000128652 |
| STAT6 | ENSG00000166888 |
| TFAP2A | ENSG00000137203 |
| ZBTB26 | ENSG00000171448 |
| ZNF263 | ENSG00000006194 |
| ETV4 | ENSG00000175832 |
| BARHL1 | ENSG00000125492 |
| BARHL2 | ENSG00000143032 |
| MNX1 | ENSG00000130675 |
| LMX1A | ENSG00000162761 |
| PRRX1 | ENSG00000116132 |
| LMX1B | ENSG00000136944 |
| HIC2 | ENSG00000169635 |
| NRF1 | ENSG00000106459 |
| SF1 | ENSG00000168066 |
| OCT6 | ENSG00000185668 |
| RORC | ENSG00000143365 |
| NR2C2 | ENSG00000177463 |
| NFATC2 | ENSG00000101096 |
| NFATC1 | ENSG00000131196 |
| NFATC4 | ENSG00000100968 |
| SOX2 | ENSG00000181449 |
| NFAT5 | ENSG00000102908 |
| ZNF189 | ENSG00000136870 |
| SOX13 | ENSG00000143842 |
| MZF1 | ENSG00000099326 |
| PRDM10 | ENSG00000170325 |
| ZNF652 | ENSG00000198740 |
| ZNF382 | ENSG00000161298 |
| GRHL2 | ENSG00000083307 |
| PRDM4 | ENSG00000110851 |
| FOXA1 | ENSG00000129514 |
| FOXP3 | ENSG00000049768 |
| FOXP1 | ENSG00000114861 |
| NFYA | ENSG00000001167 |
| SPDEF | ENSG00000124664 |
| NR5A1 | ENSG00000136931 |
| RORA | ENSG00000069667 |
| HNF6 | ENSG00000169856 |
| RELB | ENSG00000104856 |
| ESRRA | ENSG00000173153 |
| ELF5 | ENSG00000135374 |
| ESRRB | ENSG00000119715 |
| ZKSCAN1 | ENSG00000106261 |
| CTCFL | ENSG00000124092 |
| OSR1 | ENSG00000143867 |
| MYB | ENSG00000118513 |
| ZBTB32 | ENSG00000011590 |
| NR1H4 | ENSG00000012504 |
| SOX21 | ENSG00000125285 |
| SRY | ENSG00000184895 |
| SOX10 | ENSG00000100146 |
| SOX4 | ENSG00000124766 |
| MEF2C | ENSG00000081189 |

| **Table S6.** Identified TFs with potential molecular function  in promoters of SE-regulated genes in each cluster. | | | | | | | | | | | | | |
| --- | --- | --- | --- | --- | --- | --- | --- | --- | --- | --- | --- | --- | --- |
| **symbol** | **ENSEMBL** |  |  |  |  |  |  |  |  |  |  |  |  |
| MSANTD3 | ENSG00000066697 | |  |  |  |  |  |  |  |  |  |  |  |
| BATF3 | ENSG00000123685 | |  |  |  |  |  |  |  |  |  |  |  |
| FOS | ENSG00000170345 | |  |  |  |  |  |  |  |  |  |  |  |
| FOSL1 | ENSG00000175592 | |  |  |  |  |  |  |  |  |  |  |  |
| JUN | ENSG00000177606 | |  |  |  |  |  |  |  |  |  |  |  |
| PBX1 | ENSG00000185630 | |  |  |  |  |  |  |  |  |  |  |  |
| JDP1 | ENSG00000108176 | |  |  |  |  |  |  |  |  |  |  |  |
| SPI1 | ENSG00000066336 | |  |  |  |  |  |  |  |  |  |  |  |
| HOXB5 | ENSG00000120075 | |  |  |  |  |  |  |  |  |  |  |  |
| PRDM14 | ENSG00000147596 | |  |  |  |  |  |  |  |  |  |  |  |
| HOXA2 | ENSG00000105996 | |  |  |  |  |  |  |  |  |  |  |  |
| HOXB4 | ENSG00000182742 | |  |  |  |  |  |  |  |  |  |  |  |
| HOXD3 | ENSG00000128652 | |  |  |  |  |  |  |  |  |  |  |  |
| STAT6 | ENSG00000166888 | |  |  |  |  |  |  |  |  |  |  |  |
| TFAP2A | ENSG00000137203 | |  |  |  |  |  |  |  |  |  |  |  |
| ZBTB26 | ENSG00000171448 | |  |  |  |  |  |  |  |  |  |  |  |
| ZNF263 | ENSG00000006194 | |  |  |  |  |  |  |  |  |  |  |  |
| ETV4 | ENSG00000175832 | |  |  |  |  |  |  |  |  |  |  |  |
| BARHL1 | ENSG00000125492 | |  |  |  |  |  |  |  |  |  |  |  |
| BARHL2 | ENSG00000143032 | |  |  |  |  |  |  |  |  |  |  |  |
| MNX1 | ENSG00000130675 | |  |  |  |  |  |  |  |  |  |  |  |
| LMX1A | ENSG00000162761 | |  |  |  |  |  |  |  |  |  |  |  |
| PRRX1 | ENSG00000116132 | |  |  |  |  |  |  |  |  |  |  |  |
| LMX1B | ENSG00000136944 | |  |  |  |  |  |  |  |  |  |  |  |
| HIC2 | ENSG00000169635 | |  |  |  |  |  |  |  |  |  |  |  |
| NRF1 | ENSG00000106459 | |  |  |  |  |  |  |  |  |  |  |  |
| SF1 | ENSG00000168066 | |  |  |  |  |  |  |  |  |  |  |  |
| OCT6 | ENSG00000185668 | |  |  |  |  |  |  |  |  |  |  |  |
| RORC | ENSG00000143365 | |  |  |  |  |  |  |  |  |  |  |  |
| NR2C2 | ENSG00000177463 | |  |  |  |  |  |  |  |  |  |  |  |
| NFATC2 | ENSG00000101096 | |  |  |  |  |  |  |  |  |  |  |  |
| NFATC1 | ENSG00000131196 | |  |  |  |  |  |  |  |  |  |  |  |
| NFATC4 | ENSG00000100968 | |  |  |  |  |  |  |  |  |  |  |  |
| SOX2 | ENSG00000181449 | |  |  |  |  |  |  |  |  |  |  |  |
| NFAT5 | ENSG00000102908 | |  |  |  |  |  |  |  |  |  |  |  |
| ZNF189 | ENSG00000136870 | |  |  |  |  |  |  |  |  |  |  |  |
| SOX13 | ENSG00000143842 | |  |  |  |  |  |  |  |  |  |  |  |
| MZF1 | ENSG00000099326 | |  |  |  |  |  |  |  |  |  |  |  |
| PRDM10 | ENSG00000170325 | |  |  |  |  |  |  |  |  |  |  |  |
| ZNF652 | ENSG00000198740 | |  |  |  |  |  |  |  |  |  |  |  |
| ZNF382 | ENSG00000161298 | |  |  |  |  |  |  |  |  |  |  |  |
| GRHL2 | ENSG00000083307 | |  |  |  |  |  |  |  |  |  |  |  |
| PRDM4 | ENSG00000110851 | |  |  |  |  |  |  |  |  |  |  |  |
| FOXA1 | ENSG00000129514 | |  |  |  |  |  |  |  |  |  |  |  |
| FOXP3 | ENSG00000049768 | |  |  |  |  |  |  |  |  |  |  |  |
| FOXP1 | ENSG00000114861 | |  |  |  |  |  |  |  |  |  |  |  |
| NFYA | ENSG00000001167 | |  |  |  |  |  |  |  |  |  |  |  |
| SPDEF | ENSG00000124664 | |  |  |  |  |  |  |  |  |  |  |  |
| NR5A1 | ENSG00000136931 | |  |  |  |  |  |  |  |  |  |  |  |
| RORA | ENSG00000069667 | |  |  |  |  |  |  |  |  |  |  |  |
| HNF6 | ENSG00000169856 | |  |  |  |  |  |  |  |  |  |  |  |
| RELB | ENSG00000104856 | |  |  |  |  |  |  |  |  |  |  |  |
| ESRRA | ENSG00000173153 | |  |  |  |  |  |  |  |  |  |  |  |
| ELF5 | ENSG00000135374 | |  |  |  |  |  |  |  |  |  |  |  |
| ESRRB | ENSG00000119715 | |  |  |  |  |  |  |  |  |  |  |  |
| ZKSCAN1 | ENSG00000106261 | |  |  |  |  |  |  |  |  |  |  |  |
| CTCFL | ENSG00000124092 | |  |  |  |  |  |  |  |  |  |  |  |
| OSR1 | ENSG00000143867 | |  |  |  |  |  |  |  |  |  |  |  |
| MYB | ENSG00000118513 | |  |  |  |  |  |  |  |  |  |  |  |
| ZBTB32 | ENSG00000011590 | |  |  |  |  |  |  |  |  |  |  |  |
| NR1H4 | ENSG00000012504 | |  |  |  |  |  |  |  |  |  |  |  |
| SOX21 | ENSG00000125285 | |  |  |  |  |  |  |  |  |  |  |  |
| SRY | ENSG00000184895 | |  |  |  |  |  |  |  |  |  |  |  |
| SOX10 | ENSG00000100146 | |  |  |  |  |  |  |  |  |  |  |  |
| SOX4 | ENSG00000124766 | |  |  |  |  |  |  |  |  |  |  |  |
| MEF2C | ENSG00000081189 | |  |  |  |  |  |  |  |  |  |  |  |

| **Table S7** The 3-TF index values and 22-gene glycolysis signature in CCLE | | |
| --- | --- | --- |
|  | 3-TF index | glycolysis_score |
| HS229T | -2.32157 | -0.11314 |
| CALU1 | -2.17263 | -0.16127 |
| SKLU1 | -2.06563 | -0.49215 |
| NCIH838 | -2.05125 | 0.124136 |
| HCC2108 | -2.04329 | -0.83849 |
| HS618T | -1.65164 | 0.083669 |
| SW1573 | -1.58121 | 0.170294 |
| RERFLCAD2 | -1.57636 | -0.63474 |
| NCIH2085 | -1.55215 | -0.48353 |
| NCIH2405 | -1.50314 | 0.104868 |
| A427 | -1.37144 | -0.56323 |
| NCIH650 | -1.19106 | 0.54267 |
| HCC461 | -1.05001 | -0.94466 |
| NCIH2073 | -0.98627 | 0.390687 |
| NCIH23 | -0.90988 | -0.47595 |
| CAL12T | -0.90524 | -0.6939 |
| RERFLCMS | -0.90111 | -0.41919 |
| HCC364 | -0.81482 | 0.299971 |
| NCIH1650 | -0.76838 | -0.57025 |
| NCIH1568 | -0.7473 | -0.41231 |
| NCIH2291 | -0.61107 | -0.64394 |
| NCIH1435 | -0.57923 | -0.05799 |
| ABC1 | -0.56961 | -0.45064 |
| HCC827 | -0.48804 | 0.167231 |
| PC14 | -0.47473 | 0.206883 |
| LXF289 | -0.4743 | -0.00459 |
| NCIH1755 | -0.47128 | 0.191552 |
| NCIH2228 | -0.41459 | 0.125547 |
| A549 | -0.36749 | -0.64661 |
| NCIH2023 | -0.29853 | -0.09089 |
| NCIH1693 | -0.29829 | -0.8534 |
| HOP62 | -0.29745 | -0.57593 |
| MORCPR | -0.28797 | 0.24185 |
| NCIH1373 | -0.28367 | 0.129074 |
| HCC1171 | -0.28155 | 0.589088 |
| NCIH1838 | -0.21479 | -0.60744 |
| HCC44 | -0.18753 | -0.07735 |
| NCIH1563 | -0.18326 | -0.24581 |
| HCC78 | -0.17679 | 0.486306 |
| NCIH2009 | -0.17108 | 0.919728 |
| NCIH358 | -0.11749 | 0.546406 |
| RERFLCAD1 | -0.08683 | 0.371632 |
| EKVX | -0.05069 | -0.0596 |
| NCIH2030 | -0.03893 | 0.840595 |
| NCIH1793 | -0.00643 | 0.712621 |
| NCIH3255 | 0.005068 | 0.487057 |
| NCIH2087 | 0.006664 | 0.742107 |
| NCIH1944 | 0.007318 | -0.03603 |
| HCC827GR5 | 0.012764 | 0.128019 |
| NCIH1623 | 0.025887 | -0.5535 |
| PC9 | 0.044311 | 0.42484 |
| NCIH1666 | 0.06215 | 0.184813 |
| NCIH322 | 0.070418 | -0.52678 |
| NCIH1355 | 0.078024 | -0.73191 |
| HCC1833 | 0.091382 | 0.553234 |
| NCIH1573 | 0.097434 | -0.47866 |
| NCIH1648 | 0.133362 | 0.461243 |
| RERFLCKJ | 0.152774 | -0.30542 |
| NCIH1792 | 0.163039 | 0.522674 |
| HCC515 | 0.174171 | -0.42927 |
| NCIH1734 | 0.180985 | 0.340884 |
| NCIH1819 | 0.203992 | -0.97805 |
| NCIH2342 | 0.206131 | -0.26571 |
| NCIH2347 | 0.206654 | -0.34974 |
| CALU3 | 0.223293 | -0.61091 |
| HCC4006 | 0.273328 | 0.367533 |
| NCIH1975 | 0.277257 | 0.279546 |
| DV90 | 0.372494 | 0.719157 |
| NCIH1781 | 0.416891 | -0.10118 |
| NCIH1395 | 0.433413 | 0.543881 |
| NCIH441 | 0.485893 | 0.970041 |
| NCIH2122 | 0.501476 | 0.002019 |
| NCIH1437 | 0.504322 | 0.213916 |
| NCIH1651 | 0.511588 | 0.530477 |
| NCIH2077 | 0.729956 | 0.928625 |
| HCC2935 | 0.746644 | 0.313986 |
| NCIH522 | 0.880074 | -1.02996 |
| NCIH3122 | 0.942482 | 0.428827 |

**
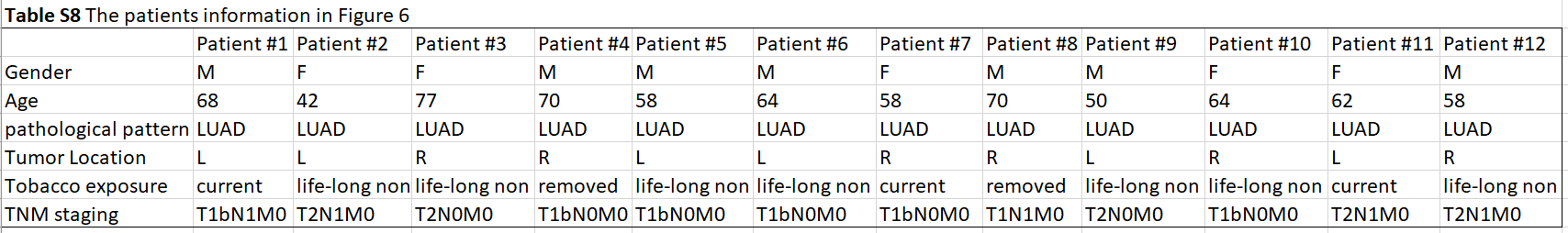
**
